# Metabolic engineering of cucurbitacins in *Cucurbita pepo* hairy roots

**DOI:** 10.3389/fpls.2022.1021907

**Published:** 2022-12-05

**Authors:** Aldo Almeida, Lemeng Dong, Theis H. Thorsen, Morten H. Raadam, Bekzod Khakimov, Natalia Carreno-Quintero, Sotirios C. Kampranis, Søren Bak

**Affiliations:** ^1^ Department of Plant and Environmental Sciences, University of Copenhagen, Frederiksberg, Denmark; ^2^ Swammerdam Institute for Life Sciences, University of Amsterdam, Amsterdam, Netherlands; ^3^ Department of Food Science, University of Copenhagen, Frederiksberg, Denmark; ^4^ Innovation for Crops, Keygene, N.V., Wageningen, Netherlands

**Keywords:** Rhizobium rhizogenes, triterpenoids, Acyl transferase, P450, Iberis amara, Cucurbita pepo, Ecballium elaterium, Cucumis sativus

## Abstract

In this paper we show that metabolic engineering in *Cucurbita pepo* hairy roots can be used to both effectively increase and modify cucurbitacins. Cucurbitacins are highly-oxygenated triterpenoids originally described in the Cucurbitaceae family, but have since been found in 15 taxonomically distant plant families. Cucurbitacin B, D, E and I are the most widespread amongst the Cucurbitaceae and they have both important biological and pharmacological activities. In this study *C. pepo* hairy roots were used as a platform to boost production and alter the structures of the afore mentioned cucurbitacins by metabolic engineering to potentially provide new or more desirable bioactivities. We report that the ability to induce cucurbitacin biosynthesis by basic Helix-Loop-Helix transcription factors is partially conserved within the Cucurbitaceae and therefore can potentially be used as a biotechnological tool to increase cucurbitacins in several genera of this family. Additionally, overexpression of a novel acyltransferase from cucurbitacin producing *Iberis amara* generates a hitherto undescribed acetylation at the C3-hydroxyl group of the cucurbitadienol backbone. While overexpression of the cytochromes P450 *CsCYP88L2* and *McCYP88L7* from *Cucumis sativus* and *Momordica charantia* (respectively), results in accumulation of new spectral feature as revealed by High resolution liquid chromatography mass spectroscopy analysis; the m/z of the new peak supports it might be a cucurbitacin hydroxylated at the C19 position in *C. pepo* hairy roots. Finally, this paper is a case study of how hairy roots can be used to metabolically engineer and introduce novel modifications in metabolic pathways that have not been fully elucidated.

## 1 Introduction

The aim of this paper was to explore the potential of hairy roots as a production platform for cucurbitacins using metabolic engineering mediated by overexpression of transcription factors and candidate genes. Cucurbitacins are plant specialized metabolites initially developed in the plant for herbivore deterrence (Ferguson and Metcalf, 1985; Dinan et al., 1997), but they have also been shown to have important pharmaceutical bioactivities such as anti-cancer, anti-inflammatory and anti-diabetic activities (Tan et al., 2008; Abdelwahab et al., 2011; Rios et al., 2012). Structurally they are a diverse type of triterpenoids; they are categorized into 17 groups, named from A to T, according to their great variety of functional groups and side-chains (Kaushik et al., 2015). It is this diversity in cucurbitacin structures that impact functionality, and it has been reported that even single modification on a triterpenoid backbone can have great impact on the structure-activity relationships (Sun et al., 2005; Almeida et al., 2020). Cucurbitacins were initially discovered in the Cucurbitaceae family but have now been shown to exist in 15 taxonomically distant families of plants, including the brassicaceae *Iberis amara* (Chen et al., 2005), indicating that the ability to biosynthesize cucurbitacins has evolved more than once in the plant kingdom (Dong et al., 2021). Cucurbitacins may also affect actin polymerization, accordingly they are also cytotoxic to normal cells, and excess amounts cause vomiting in mammals (Wattj and Breyer-brandwijk, 1962), which limits their pharmacological use. Accordingly, introduction of novel modifications in cucurbitacins could generate bioactive cucurbitacins with novel or improved pharmacological properties; however, this approach is limited due to only partial elucidation of genes encoding for enzymes in the cucurbitacin pathway.

**Figure 1:**
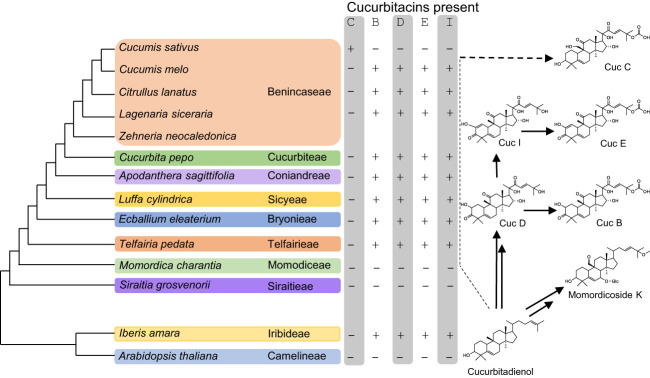
Cucurbitacins B, D, E and I are the most widespread amongst the Cucurbitaceae. On the left is a representation of the phylogenetic relationships within the Cucurbitaceae and Brassicaceae families. The presence of major types of cucurbitacins in each tribe is indicated by a + sign whilst the absence by a – sign. The right shows that cucurbitacins B, D, E and I first appear in members of the Telfairieae up to the Benincaseae tribe, but are absent in the Momodiceae tribe and *Cucumis sativus*. Within Brassicecea only *Iberis amara* is known to accumulate cucurbitacins.

**Figure 2:**
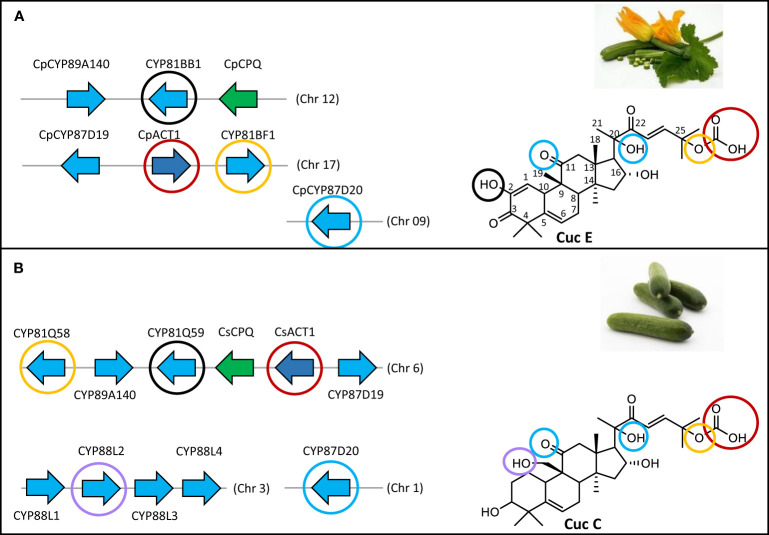
The main cucurbitacin biosynthetic cluster in *Cucurbita pepo* is split in two chromosomes. **(A)** Chromosomal organization of cucurbitacin biosynthetic genes in *C. pepo*. Modifications on the cucurbitadienol backbone performed by each gene are highlighted in distinct colored circles. **(B)** Chromosomal organization of cucurbitacin biosynthetic genes in *Cucumis sativus*. Modifications on the cucurbitadienol backbone performed by each gene are highlighted in distinct colored circles. CYP81Q58 catalyzes the C25 hydroxylation; CYP81Q59 the C2 hydroxylation; CpCPQ the first committed step in cucurbitacin biosynthesis making cucurbitadienol backbone; ACT1 transfer an acetyl group to the C25 hydroxyl, CYP87D20 is a C11 carboxylase and hydroxylates the C20 position, CYP88L2 hydroxylates the C19 position.

Cucurbitadienol is the backbone scaffold of cucurbitacins and can be oxidized at several positions, often multiple times, leading to highly oxygenated triterpenoid structures. Cucurbitacin B (CucB), D (CucD), E (CucE) & I (CucI) are the most widespread amongst the Cucurbitaceae (**Figure 1**) (David and Vallance, 1955; Rehm et al., 1957). Due to the structural complexity of cucurbitacins, their biosynthetic pathway after the initial cyclization step has only been partially elucidated. Five steps in the Cuc E pathway have been identified in members of the Benincaseae tribe, one of the 15 cucurbitaceous tribes, and found to reside in a metabolic gene cluster (Shang et al., 2014) (**Figure 2**). Four of the characterized steps are performed by Cytochromes P450 (P450s): CYP88L2 hydroxylates the C19 position of cucurbitadienol, while CYP87D20 oxidizes the C20 and C11 positions by adding a hydroxyl and carbonyl group to these carbons, respectively; CYP81Q58 hydroxylates the C25 position of cucurbitadienol and they CYP81Q59, while inactive in cucumber, paralogs of this P450 in other members of the Benincaseae tribe hydroxylate the C2 position (**Figure 2A**). The final characterized gene in the cucurbitacin E pathway encodes an acyl transferase which acetylates the C25 hydroxyl group performed by the CYP81Q58 (**Figure 2A**). Despite these advances, at least four steps in the cucurbitacin E pathway remain uncharacterized: the oxidation of the C3 hydroxyl to a carbonyl, introduction of the carbonyl at the C22 position, hydroxylation of the C16 position and formation of the C1-C2 double bond (**Figure 2A**). Furthermore, six orthologous basic helix-loop-helix (bHLH) transcription factors (TFs) that induced the expression of Cucurbitacins biosynthetic genes in the Benincaseae tribe in an organ-specific manner have been described (Shang et al., 2014; Zhou et al., 2016); these bHLH TFs are differentially expressed in different organs. Additionally, cucurbitacin profiles differ within species of the Cucurbitaceae; i.e, Cucurbitacins from *Momordica charantia* (Momordica) are not hydroxylated at the cucurbitadienol C16 position, but are oxidized at the C19 position. The C19 hydroxylation is not observed in species of the Cucurbiteae like *Cucurbita pepo* (squash) nor in most species of the Benincaseae, with the exception of *Cucumis sativus* (cucumber) (**Figure 1**). We recently showed that the cucurbitacin pathway evolved independently in *Iberis amara* of the Brassicaceae (Dong et al., 2021). This convergent evolution extends the existing limited molecular toolbox of genes from Curcurbitaceae species for metabolic engineering of cucurbitacins.

Many Cucurbitaceae species accumulate cucurbitacins in the roots, accordingly, this study explored if hairy roots are suitable targets for cucurbitacin biosynthesis induction and metabolic engineering. Hairy roots (HRs) are induced when *Rhizobium rhizogenes* transforms a set of open reading frames (ORFs) and root loci (rol) genes contained in the hairy root inducing plasmid into a wounded plant cell; the ORFs/rol genes participate in the proliferation of neoplastic roots and root hairs. Aside from the advantage of growing on phytohormone-free media, HRs are considered stable, with the capacity to grow indefinitely and thus can be contained and potentially scaled up. In addition, the use of binary vector systems can take advantage of the *R. rhizobium* DNA-integration mechanism to also deliver genes of interest into the genome of the host plant. Therefore, HRs can be especially useful for metabolic engineering of phytochemicals located in the roots for which the entire pathway has not been elucidated, such as for cucurbitacins.

We have previously shown that HRs from squash can be developed on agar plates and accumulate cucurbitacins. Furhtermore, we have reported that the approach of overexpressing squalene epoxidases e.g. CpSQE2 and OsSQE2 from squash and *Ononis spinosa* can increase the production of cucurbitacins and α-onocerin, respectively, by increasing abundance and availability of the precursor 2,3-oxidosqualene (Almeida et al., 2018; Dong et al., 2018). In this paper, squash HRs were established as a platform for metabolic engineering of cucurbitacins. This paper also reports that the ability to induce cucurbitacin biosynthesis by basic Helix-Loop-Helix transcription factors is partially conserved in the Cucurbitaceae and serves as a cucurbitacin-metabolic-engineering switch for this family, that overexpression of a novel acyltransferase from *I. amara* generates an undescribed acetylation at the C3-hydroxyl group of the cucurbitadienol backbone, and that overexpression of *CYP88L2* and *CYP88L7* from *Cucumis sativus* and *Momordica charantia*, respectively, results in accumulation of cucurbitacins hydroxylated at the C19 postion which is not normally observed in squash.

## 2 Materials and methods

### 2.1 Cloning of candidate genes

Total RNA was isolated from *Cucurbita pepo* cultivar golden glory and *Cucumis sativus* var. beit alpha ten-day-old roots using Spectrum Plant Total RNA Kit (Sigma-Aldrich). First-strand cDNA was synthesized from total RNA of roots using the GoScriptTM Reverse transcriptase kit (Promega) following the manufacture’s instructions.

Squash orthologs of genes in the cucurbitacin cluster and pathway were searched for in the Cucurbit Genomics Database (http://cucurbitgenomics.org/) by BLASTP analysis using cucumber sequences as query. Annotations of the genomic region were corrected using FgenesH gene-finder (Solovyev et al., 2006).

Primers designed for cloning candidate genes are given in **Table S1**. Amplification was done by polymerase chain reaction (PCR) in a SensoQuest Labcycler using homemade Phusion X7 polymerase. The following program was applied: 95°C for 1 min; 30 cycles of 95°C for 20 s, 50°C for 30 s, 72°C for 1 min and 30 s; 72°C for 5 min (elongation time for CpCPQ was increased to 2 min). Gel purified amplicons were ligated to the respective cloning system: i) Gateway cloned into pDONR 207 (Invitrogen) and subsequently to the pJCV51 binary vector (VIB-UGent Center for Plant Systems Biology, Vector ID: 4_60) ii) the USER modified binary vector pEAQ : HT-DEST (ref) by USER™ Enzyme (New England Biolabs) iii) pJET 1.2/blunt vector from CloneJet PCR cloning kit (Thermo Fisher Scientific). Ligation reactions were transformed into *E. coli* competent cells using the heat shock method and successful constructs were retrieved 16 hours after. Sequences of constructs were confirmed by Sanger sequencing service (Macrogen, Europe). PJCV51 empty vector from Dong et al. (2018) was used in this study.

Two uracil-specific excision-reagent (USER) vectors with uracil, tryptophan, leucine, or histidine as auxotrophic markers were used for genomic integration. The expression plasmids pESC-URA-USER (pCfB132), pESC-LEU-USER (pCfB220), and pESC-HIS-USER (pCfB291) were gift from Prof. Irina Borodina (Technical University of Denmark, Kongens Lyngby, Denmark) and pESC-TRP-USER (pWUS) was constructed by replacement of the URA3 gene with TRP1 on pCfB132. In addition, the synthetic fragments bear flanking regions containing specific restriction sites and a generic sequence compatible for USER cloning. By applying the USER cloning technique, one or two genes of interest in a uni- or bidirectional promoter of choice were simultaneously integrated into the backbone vectors by specific annealing of the overhang created between the PCR parts and digested USER cassette (Geu-Flores et al., 2007). The CsCPR and Cp P450 genes were amplified and cloned by USER cloning into the yeast expression vectors using primers listed in **Table S1** to introduce compatible USER overhangs with the corresponding promoter part (**Table S2**) and the digested AsiSI/Nb.BsmI USER cassette of the pESC-URA/LEU/HIS/TRP-USER backbone.

Yeast integrative vectors were constructed in *Escherichia coli* cells (E. cloni^®^; Lucigen) utilizing USER cloning technique. Isolated ORFs of squash genes in the cucurbitacin cluster and pathway and *S. cerevisiae* tHMG1, ERG1, ERG12 and ERG20 were amplified by PCR using primers given in **Table S1**. Three integrative vectors (pAS1_X-3, pAS2, pAS3_X-3) (**Table S2**) were used to insert Oxidosqualene cyclases (OSCs) and terpene pathway boosting genes on site 3 of chromosome X, a region known for high gene expression with low to no impact on growth rate (Mikkelsen et al., 2012). Empty integration vectors were kindly provided by Victor Forman, University of Copenhagen, Denmark. pAS1_X-3 contains an ampicillin resistance gene (AmpR), a Kluyveromyces lactis URA3 gene and a region homologous to the insertion site, X3-DOWN, and a homologous region to pAS2. pAS2 contains homologous regions to pAS1_X-3 and pAS3_X-3. pAS3_X-3 contains homologous regions to pAS2 and the integration site X3-UP. PCR fragments were USER cloned into AsiSI/Nb.BsmI digested empty vectors with PCR amplified GAL1/GAL10 promoters from *S. cerevisiae, S. mikae* and *S. arboricola*, to form pInt1-X (**Table S2**). All generated vectors were confirmed by sequencing.

Prior to yeast transformation, integrative vectors were linearized by digestion with *Not*I according to manufacturer’s protocol.

### 2.2 Hairy root transformation

A list of the plant species used and their providers can be found in **Table S3**.

Seeds for *Cucurbita pepo* cultivar golden glory and *Iberis umbellata* were first sterilized in a 50-ml falcon tube by washing in 30 mL of deionized water with 300 µL of Mild Cream Soap (ABENA, Denmark) and shaken for 20 min. Seeds were then rinsed in 70% ethanol for 1 min. Afterwards, seeds were submerged in 1.5% NaOCl and shaken for no more than 15 min. Subsequently, the seeds were rinsed in sterile water 6 times. The wet seeds were placed on a sterile petri dish in the sterile bench to surface dry. When the seeds were surface dried, seeds were placed on plates with ½ Murashige and Skoog media (MS) agar supplemented with 3% glucose sealed with parafilm (Merck KGaA, Darmstadt, Germany) and placed in a climate chamber. The climate chamber was from Fitotron type SGC120-H from 2016 (Weiss Technik UK Ltd., Epinal Way, Loughborough, United Kingdom) and the settings were: light at 79 uE, 25°C, 80% humidity, 16-hour photoperiod.

*Rizhobium rhizogenes* strain LBA9402 was transformed with pJCV51, pJCV51:*CpCUCbH1* through electroporation and recovered on S.O.C medium for 2 hours at 28°C before being plated on YEB agar supplemented with spectinomycin (50 µg/ml) and rifampicin (50 µg/ml). A single colony was selected for each transformation and grown overnight in liquid YEB supplemented with spectinomycin and rifampicin at 28°C under constant agitation (180 rpm). Subsequently, 50 μL of the *R. rhizogenes* suspension was placed on solid YEB medium supplemented with spectinomycin and rifampicin, and grown for two days at 28°C and colonies resuspended into PS buffer [10 mM PIPES (Sigma-Aldrich P8203)/KOH pH 6.8, 200 mM Sorbitol] supplemented with acetosyringone to a concentration of 100 μM. Finally the OD_600_ of the suspension was adjusted to 0.4.

For squash, cotyledons were transformed with *R. rhizogenes* ten days after germination. Cotyledons were inoculated by bruising with a sterile syringe needle dipped in the *R. rhizogenes* suspension. Four incisions were cut on the abaxial side of the cotyledon perpendicular to the central vein and where placed with the abaxial side down onto ½ MS (3% glucose) agar plates without antibiotics and incubated in the dark for two days. Afterwards, the inoculated cotyledons were transferred to ½ MS (3% glucose) agar plates supplemented with cefotaxim (500 µg/ml), carbenicillin (250 µg/ml) and kanamycin (50 µg/ml) and incubated under light for a week, cotyledons were subsequently transferred to new plates where cefotaxim and carbenicillin concentrations were halfed. Transformed hairy roots expressing mRFP would start to emerge after three weeks of tissue culture. These roots were excised from leaves and subcultured every two weeks with cefotaxim and carbenicillin and kanamycin. The concentrations of cefotaxim and carbenicillin were reduced 50% in each subculturing step until no antibiotic was used, nevertheless kanamycin concentration remained constant.

In the case of *I. umbellata* optimal age for transformation was optimized to be 5 weeks after germination for leaves and stems. For the leaves a similar procedure to that of squash was used, but stems were inoculated by cutting them transversally with a sterile razor blade dipped in the *R. rhizogenes* suspension. Subsequent cultivation were performed the same way as described above for squash cotyledons. All incubation periods were done in the climate chamber.

### 2.3 Cultivation in temporary immersion reactors (TIR) and methyl jasmonate (MeJA) treatment

Squash HRs were grown 2 weeks in MS agar plates before being transferred to temporary immersion bioreactors (CIRAD Ltd., France) which were previoiusly sterilized by autoclaving at 121°C for 15 min. The TIR were filled with 400 ml of ½ MS media, HRs were flooded for intervals of 30 min every 8 hours for a period of 14 days. The flow rate of inlet air was 60 l/h.

For MeJ treatment, a 100mM stock solution of MeJ of 95% purity (Sigma Aldrich) was prepared in 96% ethanol and filter sterilized. After incubating HRs for 14 days in TIR, 400 µl of the MeJA stock solution was added to make a ∼100 µM MeJA treatment. In the same manner, 30 µl of MeJA sterilized stock solution were pipetted in the ½ MS agar near 14-day-old HR (square plates typically had 30 ml of agar). Samples were collected after 24 hours of adding MeJA, frozen in liquid nitrogen and stored at -70°C until further processing. All experiments in TIRs were carried out in triplicates and statistical significance was tested using one-way ANOVA (*p* < 0.05).

### 2.4 Transient expression experiments

pEAQ-HT-DEST expression vector (Sainsbury et al., 2009) constructs harboring *eGFP* and *CpCUCbH1* (described above) were transformed into *Rhizobium (Agrobacterium) tumefaciens* strain AGL1. Colonies of *A. tumefaciens* were picked in the morning and precultured in 5 ml of Luria-Bertani (LB) media supplemented with kanamycin (50 µg/ml). Afterward, 12 ml of LB media containing kanamycin was inoculated with 50 µL of the preculture and incubated at 28°C overnight without allowing OD_600_ to exceed 1.5. The cultures were subsequently centrifuged at 3000g, the resulting cell pellet resuspended in infiltration buffer (10 mM MgCl2, 10 mM MES, pH 5.6, and 100 µM acetosyringone), and the OD_600_ was adjusted to 0.4. The suspension was incubated for 1 h on a shaker at room temperature. Age of infiltration for tissues of each species was optimized: for cotyledons of squash and cucumber were infiltrated 5 days after germination and examined 5 days after infiltration, whereas cotyledons from Luffa were infiltrated 7 days after germination and examined 7 days after infiltration. In the case of *E. elaterium* leaves of 2-month-old plants were infiltrated and examined 5 days after infiltration, while for *Nicotiana benthamiana* (tabacco) leaves were infiltrated when plants were 5-weeks old and harverted 5 days after infiltration. For infiltration experiments plants were grown in soil (Pindstrup substrate no. 2) in a glasshouse with a 16-h day at 28°C and an 8-h night at 28°C. Infiltrated tissue for confocal microscopy was harvested the same day while for metabolite analysis tissues were frozen in liquid N_2_ and stored at −70°C until further processing. All experiments were carried out in triplicates and statistical significance was tested using one-way ANOVA (*p* < 0.05).

### 2.5 Gene expression analysis by qRT-PCR

Gel electrophoresis confirmed that the primers designed for qRT-PCR (**Table S4**) amplify only one amplicon. Each tissue was analyzed in triplicate, synthesizing cDNA from 1 µg of total RNA using the GoScript™ Reverse transcriptase kit (Promega), and final volume was adjusted to 10 ng/µl. All quantitative PCRs for the three primer sets were performed in the CFX384 real-time system (Bio-Rad) under the following conditions: 4 µL of PowerUp SYBR Green Master Mix (Thermo Fisher Scientific), 1 µL of forward primer (2 µm), 1 µL of reverse primer (2 µm), 1 µL of cDNA (10 ng/µl), and 1 µl of MilliQ water. The PCR conditions were initial 95°C for 5 min, followed by 40 cycles of 95°C for 15 s, 50°C for 15 s, and 72°C for 30 s.

### 2.6 Visualization of transient-expression efficiency

For visualization of *in planta* transient expression, confocal Laser Scanning Microscopy (CLSM) was done essentially as previously described (Laursen et al., 2016). Briefly, leaf discs from infiltrated tissue described above were excised, mounted in water, and observed by CLSM. Cell imaging was performed using an SP5x laser scanning confocal microscope equipped with a DM6000 microscope (Leica, Germany). Images were recorded using a 63x water immersion objective lens. Excitation/emission wavelengths were 488/500–550 nm for eGFP. The images were sequentially acquired and processed using the LAS X software (Leica).

### 2.7 Heterologous expression in *Saccharomyces cerevisiae*

The yeast strain EGY48 (Thomas and Rothstein, 1989; Ellerström et al., 1992; Ignea et al., 2011) was transformed with yeast integrative vectors using the lithium acetate method (Gietz and Schiestl, 2007), generating strains EGY48-EV, EGY48-CpCPQ. Subsequently, EGY48-CpCPQ was co-transformed with plasmids pCfB220 containing the *Cucumis sativus* Cytochrome P450 Reductase (pCfB220:CsCPR) and pCfB291 containing one of the following P450s: CpCYP88L4, CYP88L2, CYP88L7, CpCYP87D19, CpCYP89A140; a full list of the strains generated is presented in **Table S4** and the description of the plasmids used is in **Table S2**. Colonies following transformation were genotyped for correct integration using PCR. For triterpenoid production, pre-cultures of the strains were grown overnight until saturation in 10 ml synthetic defined media containing 2% glucose. Then, the cells were washed twice with water and heterologous genes induced by inoculation into 10 mL synthetic defined media with 4% galactose and 2% raffinose with or without 10 mM β-methyl cyclodextrins and grown for 96 h at 30°C and 150 rpm.

### 2.8 Phylogenetic analysis

Protein-coding sequences were deduced using the Expasy translate tool (http://web.expasy.org/translate/) and manually curated. Models for the evolutionary histories for the bHLH TFs, CYP88s and ACT protein sequences were inferred separately by using the maximum-likelihood method based on the JTT matrix-based model (Jones et al., 1992). The tree with the highest log likelihood was selected (−4579.11 for the bHLH, - 6,515.95 for the CYP88 and −8,676.26 for the ACT trees, respectively). Initial trees for the heuristic search were obtained by applying the neighbor-joining method to a matrix of pairwise distances estimated using a JTT model. The trees are drawn to scale, with branch lengths measured in the number of substitutions per site. The analysis involved 21, 11 and 14 amino acid sequences for the bHLH TFs, CYP88s and ACT trees, respectively. All positions containing gaps and missing data were eliminated. In total, there were 187, 434 and 366 positions in the final data sets for the bHLH, CYP88 and ACT trees, respectively. The statistical significance of each node was tested by the bootstrap method using 1,000 iterations. The evolutionary analyses were conducted in MEGA7 (Kumar et al., 2016). Accession numbers for sequences used in phylogenetic trees are given in **Table S5**.

### 2.9 Cucurbitacin-feeding experiments in *Saccharomyces cerevisiae*

Strains EGY48-CpCPQ+CsCPR+CYP88L2 and EGY48-CpCPQ+CsCPR+CYP88L2 were grown overnight until saturation in 10 ml synthetic defined media containing 2% glucose. Then, the cells were washed twice with water and heterologous genes induced by inoculation into 10 mL synthetic defined media with 4% galactose and 2% raffinose with 10 mM β-methyl cyclodextrins. In addition, 100 µl of either 1mM Cucurbitacin B, D, E or I were added to the cultures and grown for 96 h at 30°C and 150 rpm. Additionally, production of either Cuc B or E by feeding Cuc D or I to a cultures of strain EGY48-CpACT1 grown in a similar fashion as described above was used as controls, to make sure that feeding of yeast with cucurbitacins was indeed possible. All reactions were carried out in triplicates.

### 2.10 Root phenotyping

WinRHIZO, a root image analysis software (Arsenault et al., 1995) was used for determination of primary root length, number of root tips and total root length phenotypes of HR lines. Pictures for root phenotyping were taken from HRs in agar plates 5 days after sub-culturing using a Nikon D5600 DSLR camara with an AF-P DX 18-55 VR lens (Nikon, Japan).

### 2.11 Analysis of cucurbitacins by GC-MS

For GC-MS analysis, 50 µL of extract was aliquoted into a glass insert and evaporated under vacuum. The glass inserts were sealed with air-tight magnetic lids into GC-MS vials and derivatized by the addition of 30 μL of trimethylsilyl cyanide as described before (Khakimov et al., 2013). All steps involving sample derivatization and injection were automated using a MultiPurpose Sampler (MPS; Gerstel). After reagent addition, the sample was transferred into the agitator of the MPS and incubated at 40°C for 40 min at 750 rpm. Immediately after derivatization, 1 μL of the derivatized sample was injected in splitless mode. The spilt/splitless injector port was operated at 320°C. The septum purge flow and purge flow to split vent at 2.1 min after injection were set to 3 and 15 mL min−1, respectively. The GC-MS system consisted of an Agilent 7890A GC device and an Agilent 5975C series MSD (Agilent Technologies). GC separation was performed on an Agilent HP-5MS column (30 m × 250 μm × 0.25 μm) by using hydrogen carrier gas at a constant flow rate of 1.2 ml/min. The GC oven temperature program was as follows: initial temperature, 40°C; equilibration time, 2 min; heat up to 270°C at the rate of 12°C/min; heat at the rate of 6°C/min until 310°C; and hold for 10 min. Mass spectra were recorded in the range of 50 to 700 mass-to-charge ratio with a scanning frequency of 2.2 scans/s, and the MS detector was switched off during the first 20 min of the run, since all targeted molecules eluted after this retention time. The transfer line, ion source, and quadrupole temperatures were set to 290°C, 230°C, and 150°C, respectively. The mass spectrometer was tuned according to the manufacturer’s recommendation by using perfluorotributylamine. The MPS and GC-MS devices were controlled using vendor software Maestro (Gerstel).

### 2.12 Analysis of cucurbitacins by LC-QToF-MS

First, 100 mg of frozen powdered material were extracted with 600 µL of methanol (containing 50 µM of protopanaxatriol as internal standard) in a 1.5 mL vials with screw cap. Vials were ultrasonicated for 20 min at room temperature, the vials were then centrifuged for 10 min at 13,000 rpm and the supernatant was filtered through 0.22 µm centrifugal filters (UFC30GV, Merck Millipore). All treatments consisted of at least three replicates (specific replicate numbers are specified in figure legends). Statistical significance was tested usin one-way ANOVA (*p* < 0.05).

For extraction of triterpenoids from yeast cultures incubated with β-methyl cyclodextrin, 10 mL of culture was extracted using 10 mL ethyl acetate. The mixture was vortexed for 2 minutes, then centrifuged at 3,000g for 10 minutes and the organic phase collected, evaporated under vacuum and the residues were resuspended in 200µL of methanol. For extraction of triterpenoids from yeast cultures not containing β-methyl cyclodextrin, the cell pellet from 10 ml culture was resuspended in 500µL 10% KOH, 80% EtOH and saponified for 2 hours at 70°C. The samples were then extracted three times with 500µL hexane, which was pooled, evaporated under vacuum and the residues resuspended in 200µL of Methanol.

A Dionex UltiMate™ 3000 RS UPLC system from Thermo Scientific™ (Waltham, MA USA) was used and equipped with a KINETEX^®^ XB-C18 column (2.1 mm × 100 mm, 1.7 µm, Phenomenex). Mobile phase A was prepared using MilliQ water and 0.05% formic acid, whereas mobile phase B consisted of acetonitrile and 0.05% formic acid. The gradient was t = 0 min, 5% B; t = 40 min, 100% B; t = 45 min, 100% B; t = 46 min, 5% B; t = 50 min, 5% B. The chromatographic run lasted 50 min with a flow rate of 0.3 ml/min. For detection, a Bruker compact™ QTOF mass spectrometer (Bremen, Germany) was used and operated in negative mode. The mass spectrometer’s settings were: dry gas flow rate 8 L min−1 at 220°C, capillary 4500 V, collision energy 7 eV and collision RF 500Vpp, transfer energy 100 µs, pre-Pulse storage 5µs. The QTOF operated with the mass range set from 50 to 1200 m/z and was calibrated with sodium formate clusters at the beginning of every injection. The injection volume was 5 μL per sample. Acquisition of LC-MS data was performed under Bruker DataAnalysis 4.3. Quantification of cucurbitacins was performed in more than three lines for each HR construct/treatment. Calibration curves with cucurbitacins B,D,E&I (Extrasynthese, Lyon, France) were made for quantification of cucurbitacin production in hairy roots lines.

Suggested chemical structures for the putative cucurbitacins in **Figures 7**; **S7**. have a metabolite identification confidence level of 4, according to the Metabolomics Standards Initiatives (Sumner et al., 2007; Schrimpe-Rutledge et al., 2016) and was achieved by molecular formula generated from accurate mass spectrometry data (± 5 ppm) and MS/MS fragmentation pattern.

## 3 Results

### 3.1 Growth in temporary immersion reactors increases biomass and cucurbitacin content of HR as compared to agar plates and liquid cultures

**Figure 3:**
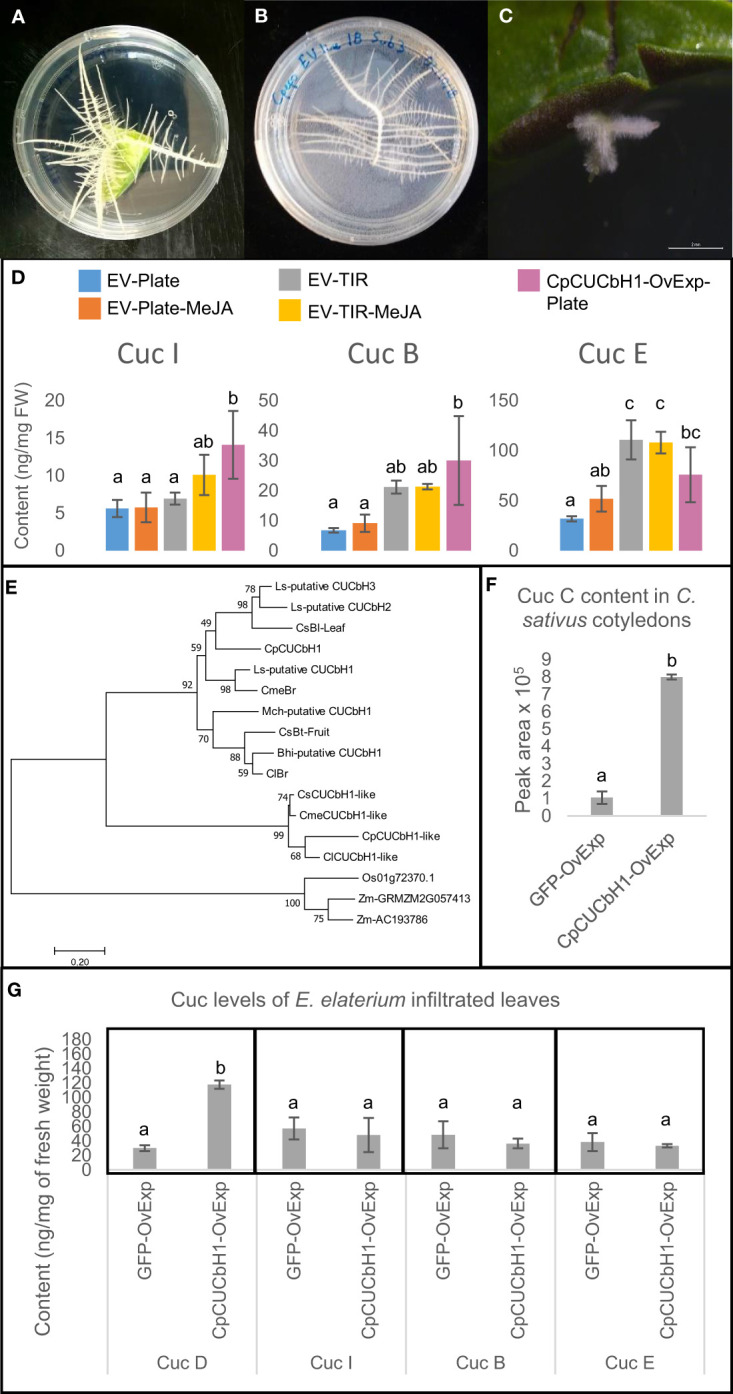
Cucurbitacin production in *C.ucurbita pepo* hairy roots can be increased by overexpression of *C.pCUCbH1* and cultivation methods. **(A)** Hairy roots emerging from *C. pepo* cotyledon 2 weeks after transformation. **(B)** *C. pepo* hairy roots propagation on agar plate. **(C)** hairy roots emerging from *Iberis umbellata* are smaller and grow slower than *C. pepo* hairy roots, the picture was taken 8 weeks after transformation. **(D)** Comparison of Cucurbitacin I, B and E content between *C. pepo* hairy root lines overexpressing different gene constructs and grown under different conditions i.e. agar plates (*n* = 5), temporary immersion reactors (*n* = 3). CpCUCbH1-OvExp lines were grown in plates (*n* = 3). **(E)** Phylogenetic tree of basic Helix Loop Helix transcription factors regulating cucurbitacin production in the Cucurbitaceae. Accession numbers for genes are in **Table S5**. Sequences of bHLH clade II transcription factors from Zea mays and Oriza sativa were used as an outgroup. **(F)** Relative abundance of Cucurbitacin C in *C.ucumis sativus* cotyledons infiltrated with different constructs (*n* = 3). **(G)** Concentration of Cucurbitacin D, I, B and E in *Ecballium elaterium* leaves infiltrated with constructs overexpressing GFP or CUCbH1 (*n* = 3). EV- empty vector control, TIR- grown in temporary immersion reactors, MeJA- induced with Methyl jasmonate, CpCUCbH1- samples overexpressing transcription factor CpCUCbH1, GFP- Green Fluorescent Protein control. Error bars are standard deviation. Statistical significance was tested using one-way ANOVA (*p* < 0.05).

Our previous work established a HR transformation protocol for Squash (Dong et al., 2018) based on their growth on agar plates. Recently we showed that *I. amara* produce Cucs B,D,E&I in all organs (Dong et al., 2021), suggesting that Iberis species could be an alternative to squash HR for metabolic engineering of cucurbitacins. Hence, in this study a HR transformation protocol for *Iberis umbellata* was developed, to enable a comparison between *I. umbellate* and squash as HR platforms for metabolic engineering of cucurbitacins (**Figures 3A-C**). Due to the ∼7-fold higher transformation efficiency and shorter time taken to generate analyzable HRs, further work was carried out in squash HRs as opposed to *I. umbellata* HRs (table in **Figure S1**).

To establish a reproducible and scalable production platform, growth of squash HRs on agar plates, in liquid culture and in temporary immersion reactors (TIRs) was compared. In terms of cucurbitacin production, metabolite profiling by LC-MS showed that accumulation of Cuc E significantly increased ( ∼2-fold) in empty vector (EV) HR lines grown in TIRs as compared to those in plates but not for Cucs B&I (**Figure 3D**). Subsequent attempts to increase cucurbitacin yields of HR grown on plates and TIRs involved inducing their production with Methyl-Jasmonate (MeJA). Although the mean values appear higher for HRs grown on plates treated with MeJA (5.78 ± 1.96, 9.16 ± 2.91 and 51.98 ± 12.87 ng/mg DW for Cuc I, Cuc B and Cuc E, respectively) when compared to untreated control (5.63 ± 1.14, 6.82 ± 0.74 and 31.93 ± 2.68 ng/mg DW for Cuc I, Cuc B and Cuc E, respectively), there was no significant difference in cucurbitacin production (**Figure 3D**). Similarly, addition of MeJA did not significantly elevate cucurbitacin levels in TIRs. In terms of biomass production, HRs on plates grew slowly and were limited in space, whilst growth in liquid media inside Erlen Meyer flasks resulted in a drastic variation of biomass production within lines (**Figure S2A-C**). In summary, upscaling and reproducible production of biomass as well as higher cucurbitacin yields is best in TIRs (**Figure S2D**), probably due to facilitated aeriation, and is not affected by addition of MeJA.

### 3.2 The transcription factor CpCUCbH1 induces cucurbitacin biosynthesis in several Cucurbitaceae clades

Our previous work showed that overexpression of squash squalene epoxidases can be used to increase cucurbitacin levels in both HRs and the tabacco transient-expression systems by increasing the triterpenoid precursor 2,3-oxidosqualene (Dong et al., 2018). In this paper the approach of increasing cucurbitacins through overexpression of transcription factors was attempted.

Previously, a TF named CsBL, belonging to the clade II of the bHLH superfamily was reported to increase cucurbitacin C levels in cucumber leaves. Hence, an orthologous bHLH TF that would induce cucurbitacin accumulation in squash HRs and could be used as a tool in metabolic engineering of cucurbitacins was searched for in squash. BLASTp was used to searched the squash genome for putative orthologous sequences of the CsBL TF. This identified two sequences in the loci Cp4.1LG05g03810.1 and Cp4.1LG09g01220.1 which were 57% and 36% identical at aa level, respectively. These genes were termed *Cucurbitacin inducing bHLH transcription factor 1* (*CpCUCbH1*) and *CpCUCbH1-like*.

**Figure 4:**
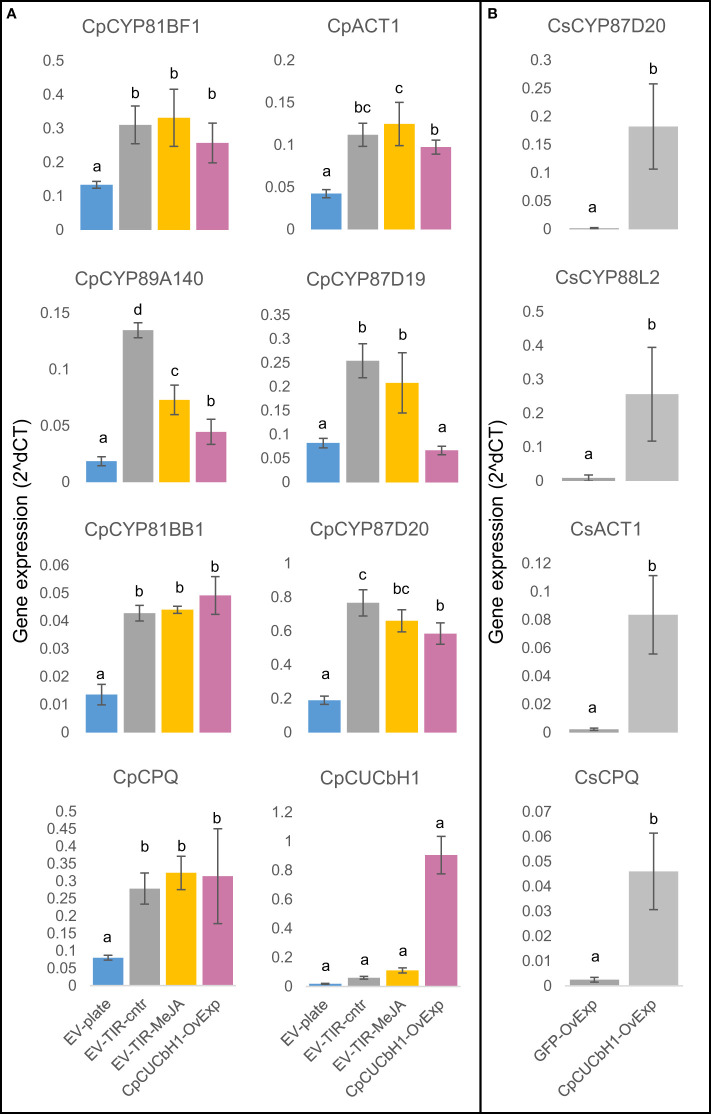
Overexpression of *C.pCUCbH1* increases expression of cucurbitacin biosynthetic genes in *C. pepo* hairy roots and *C. sativus* cotyledons. **(A)** Relative expression of selected cucurbitacin biosynthetic genes between *C. pepo* hairy root lines overexpressing *C.pCUCbH1* and grown under different conditions: *C.pCYP81BF1 (CsCYP81Q58* ortholog *C.pCYP89A140* (*C.sCYP89A140* ortholog), *C.pCYP81BB1* (CsCYP81Q59 ortholog), *C.pCPQ* (*C.sCPQ* ortholog), *C.pACT1* (*C.sACT1* ortholog), *C.pCYP87D19* (*C.sCYP87D19* ortholog), *C.pCYP87D20* (*C.sCYP87D20* ortholog), *C.pCUCbH1* (*C.sBl* ortholog). **(B)** Relative expression for selected cucurbitacin biosynthetic genes between *C. sativus* cotyledons overexpressing different constructs: *C.sCPQ, CsACT1, CsCYP87D20, CsCYP88L2*.

A phylogenetic analysis showed that CpCUCbH1 was the most likely putative ortholog of CsBL while CpCUCbH1-like formed a separate clade from these sequences (**Figure 3E**). Overexpression of *CpCUCbH*1 in squash HR grown on plates increased ∼5-fold the production of Cuc I&B, and ∼3-fold of Cuc E (**Figure 3D**). To confirm that CpCUCbH1 was the functional ortholog of CsBl, the expression levels of known genes in the cucurbitacin pathway were quantified using RT-qPCR. With the exception of CpCYP87D19, there was a significant increase in the expression of all tested genes in the *CpCUCbH1* overexpressing lines as compared to the empty vector (EV) lines (**Figure 4**). These results suggest that CpCUCbH1 is indeed an ortholog of CsBl, and that the evolution of specific TFs regulating the production of cucurbitacins occurred at least before the split of the Cucurbitae and Benincasea tribes in the Cucurbitacea family. Interestingly, although growing HR lines in TIRs increased expression of genes in the cucurbitacin cluster and pathway as compared to HRs in agar plates, growth in TIR did not increase expression of CpCUCbH1 (**Figure 4A**). This suggests there are other mechanisms besides the clade II TFs of the bHLH superfamily which may be involved in the regulation of cucurbitacin biosynthesis which respond to different environmental stimuli.

It is noteworthy that the increase of cucurbitacins in *CpCUCbH1*-overexpressing HR lines was followed by a distinctive HR phenotype of reduced lateral roots, resulting in lines without the typical fish-bone phenotype of HRs, in the end this affected the biomass produced (**Figure S3**). Hence, propagation efforts to produce enough biomass to explore the cucurbitacin production of *CpCUCbH1*-overexpressing HR lines in TIRs were not attempted due to the poor growth reported by these lines.

An orthologous bHLH TF sequence of Momordica grouping with CsBl and CpCUCbH1 in the phylogetic tree (**Figure 3E**) made us suspect that the bHLH induction of cucurbitacins could be widespread in the Cucurbitaceae. To determine if CpCUCbH1 could be used as a biotechnological tool to induce cucurbitacins in different species of the Cucurbitaceae, cotyledons or leaves of several Cucurbitaceae species were transiently infiltrated, and cucurbitacin levels, and expression levels of selected cucurbitacin biosynthetic genes were measured. To test if the squash bHLH could induce cucurbitacin biosynthesis in other Cucurbitaceae species, *CpCUCbH1* was first transiently expressed in cucumber cotyledons. Since genes in the cucurbitacin biosynthesis pathway of cucumber have been reported, their expression could be monitored by qPCR compared to cucurbits in deeper Cucurbitaceae lineages that exhibit a lack of genetic resources. overexpression of CpCUCbH1 resulted in ~8 fold higher accumulation of Cuc C (**Figure 3F**) and higher expression of genes in the Cuc C pathway (**Figure 4B**). Having confirmed this, we next tested induction of cucurbitacins in deeper Cucurbitaceae lineages. *Luffa cylindrica* (Luffa) is a member of the Sicyeae tribe which split from the Cucurbitae (**Figure 1**) 39 ± 3 Million Years ago (MY) (Schaefer et al., 2009) and produces Cucs B,D,E&I in leaves (Rehm et al., 1957); cotyledons of Luffa were successfully infiltrated and constructs were overexpressed, as observed from fluorescence of the GFP control constructs (**Figure S4A-D**). However, cotyledons of Luffa did not accumulate Cuc B,D,E&I, and overexpression of *CpCUCbH1* did not induce production of cucurbitacins (**Figure S4E**). This incapacity of CpCUCbH1 to induce cucurbitacin biosynthesis was also observed for squash cotyledons (**Figure S4F**).

Finally, transient-expression experiments were performed on *Ecballium elaterium* to test if CpCUCbH1 would also increase cucurbitacin production in species of deeper phylogenetic clades in the Cucurbitaceae. *Ecballium elaterium* is a cucurbit which accumulates Cucs B,D,E&I (David and Vallance, 1955) in its leaves and belongs in the Bryonieae tribe, a tribe that split from the Cucurbitae 41 ± 3 Million Years ago (MY) (Schaefer et al., 2009). The transient-infiltration of *E. elaterium* leaves experiments for overexpression of CpCUCbH1 significantly increased levels of Cuc D ∼4-fold but not of Cucs B,E&I (**Figure 3G**). Induction of cucurbitacins in the deeper lineage of the Momordiceae was also attempted, but unfortunately leaves of Momordica proved difficult to infiltrate.

These results suggest that induction of cucurbitacin biosynthesis by clade II bHLH TF may require additional factors and is partially conserved in the Cucurbitaceae. Nevertheless, here it is shown that *CpCUCbH1* overexpression can be used as a tool to induce cucurbitacins in selected clades of this family.

### 3.3 Modifying cucurbitacins through metabolic engineering

#### 3.3.1 An acyl transferase from *Iberis amara* produces undescribed C3 acetylated cucurbitacins

**Figure 5:**
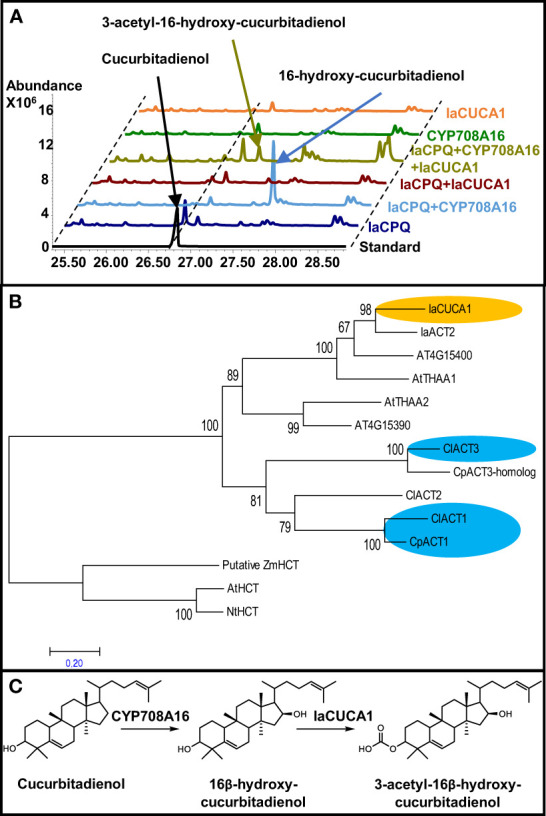
An acyltransferase from *Iberis amara* (IaCUCA1) acetylates the C3-hydroxyl position of 16-hydroxy-cucurbitadienol and has a different evolutionary origin than Cucurbitaceae acyl transferases acting on cucurbitacins. **(A)** Total ion chromatograms from GC-MS runs of *Nicotiana benthamiana* leaves infiltrated with different constructs. IaCPQ, *Iberis amara* cucurbitadienol synthase; CYP708A16, *Iberis amara* P450 making 16β-hydroxyl cucurbitadienol; IaCUCA1, *Iberis amara* acyltranferase making 3-O-acetyl-16β-hydroxyl-cucurbitadienol. **(B)** Maximum Likelihood tree constructed on deduced amino acid sequences of 14 acyl transferases aligned by ClustalW spanning 366 positions using the MEGA7 program. The statistical significance of each node was tested by the bootstrap method using 1,000 iterations. Representative names are as follows: IaCUCA1, *Iberis amara* acetyltransferase for the C3-hydroxy group of 16-hydroxy-cucurbitadienol; IaACT2, *Iberis amara* uncharacterized acyl transferase; At4G15400, *Arabidopsis thaliana* BAHD acyl transferase involved in brassinosteroid metabolism; AtTHAA1, *Arabidopsis thaliana* acetyltransferase for C15-hydroxy of 7,15-dihydroxy-16-keto-thalianol; AtTHAA2, *Arabidopsis thaliana* acetyltransferase for C3-hydroxy of different thalianol-derived compounds; At4G15390, *Arabidopsis thaliana* HXXXD-type acyl-transferase family protein; ClACT3, *Citrullus lanatus* acetyltransferase acting on C16-hydroxy of Cucs B,D,E&I; CpACT3, *Cucurbita pepo* homolog of ClACT3; ClACT2, *Citrullus lanatus* uncharacterized acyltransferase; ClACT1, *Citrullus lanatus* acetyltransferase acting on C25 hydroxyl of Cucs D&I; CpACT1, *Cucurbita pepo* functional homolog of ClACT1; the following sequences were used as an outgroup: AtHCT, Arabidopsis thaliana hydroxycinnamoyl transferase; NtHCT, Nicotiana tabacum hydroxycinnamoyl transferase; ZmHCT, Zea mays putative hydroxycinnamoyl transferase. Accession number of sequences are given in **Table S5**. **(C)** Structure and proposed biosynthesis of 3-acetyl-16-hydroxy cucurbitadienol.

We previously reported how the independent recruitment from the same gene families, I.e. OSCs and P450, lead to the convergent evolution of the cucurbitacin pathway in *I. amara* (Dong et al., 2021). Thus, we hypothesized that *I. amara* could harbor enzymes making unprecedented modifications to cucurbitacins from cucurbitacous plants. Therefore, to attempt to engineer potentially new cucurbitacins, our previously constructed transcriptome of *I. amara* was mined for candidate genes involved in modification of cucurbitacins, focusing first on acyl tranferases (ACTs). BLASTp was used to search for homologous ACTs in *I. amara* using as query the aa sequence of cucumber ACT1 (CsACT1, Csa6G088700) in the cucurbitacin gene cluster (Shang et al., 2014), as this enzyme acetylates the hydroxyl group at position C25 of Cuc B&I. This returned two sequences (Genbank: MZ695218 and MZ695219, named IaCUCA1 and IaACT2, respectively); IaACT2 showed 36% identity at amino acid level to CsACT1, while IaCUCA1 showed 56%. The function of IaCUCA1 was tested using *Rhizobium* (*Agrobacterium) tumefaciens*-mediated transient expression in tabacco. When IaCUCA1 was coexpressed with *I. amara* cucurbitadienol synthase (IaCPQ), the cucurbitadienol peak lowered in intensity but no new major peak could be detected in GC-MS (**Figure 5A**). This discrepancy could be interpreted in different ways: i) the result suggests that cucurbitadienol was not the *in planta* substrate for IaCUCA1 or ii) IaCUCA1 did act on cucurbitadienol, but the resulting triterpenoids were converted into other compounds that could not be detected with the GC-MS method used. However, when IaCUCA1 was coexpressed with IaCPQ and CYP708A16 (the *I. amara* P450 that makes 16-β-hydroxy cucurbitadienol) the intensity of the 16-β-hydroxy cucurbitadienol was lowered and a new peak could be detected (**Figure 5A**). This peak was isolated from infiltrated tobacco leaves using preperative HPLC, and the structure was identified as 3-acetyl-16-β-hydroxy-cucurbitadienol by NMR experiments (**Supplementary File 1**) (**Figure 5**). Acetylation at the C3 position of cucurbitacins has not been reported in species of the Cucurbitaceae nor in *I. amara*, making this an unexpected finding. A phylogenetic analysis of selected ACTs showed the two ACTs from *I. amara* are evolutionarily distinct from Cucurbitacea ACTs. IaCUCA1 and IaACT2 appear to have diverged from a common ancestor in the Brassicaceae which also gave rise to the clades containing AtTHAA1 and AtTHAA2 (**Figure 5B**), which both act on the triterpene thalianol of Arabidopsis (Huang et al., 2019). Thus, IaCUCA1 appears to be an acyl transferase which has a predisposition for triterpenoid scaffolds and could be used to create novel cucurbitacin structures through metabolic engineering.

Because acetylation at the C3 postion of cucbitacins from cucurbitaceous species had not been reported, metabolic engineering of cucurbitacins by overexpressing *IaCUCA1* in squash HR was attempted. Unexpectedly, only a single HR line was obtained from 120 cotyledons, which is a 0.8% transformation efficiency compared to the ∼50% efficiency normally observed for squash (**Figure S1**); in addition, this root stopped growing before being large enough for further analysis by LCMS. This suggests that IaACT is making toxic compounds that are detrimental to squash HR growth.

#### 3.3.2 Modification of Cucurbitacins by overexpression of genes in the CYP88L subfamily

**Figure 6:**
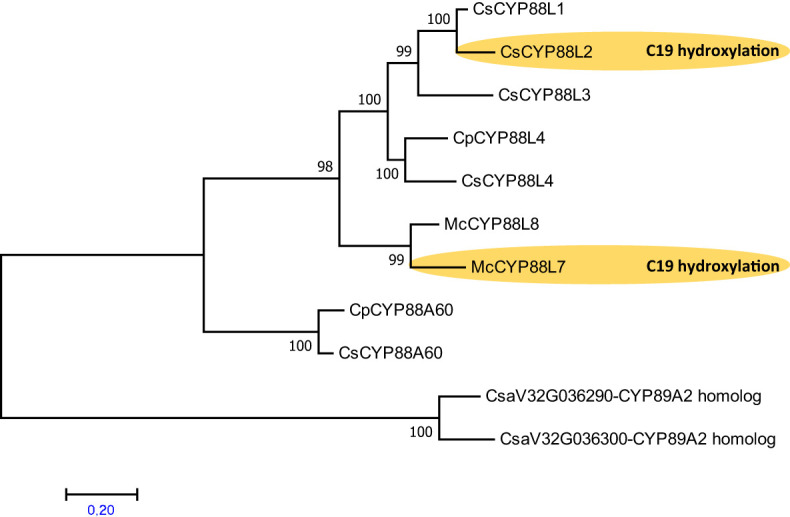
Phylogenetic analysis suggests that the P450 s the C19 hydroxylation in cucurbitadienol from *Cucumis sativus* and *Momordica charantia* evolved independently within the Cucurbitaceae. Maximum Likelihood tree constructed on deduced amino acid sequences aligned by ClustalW spanning 260 positions using MEGA7 program. The statistical significance of each node was tested by the bootstrap method using 1,000 iterations. Representative names are as follows: CsCYP88L3, *Cucumis sativus* uncharacterized P450; CsCYP88L1, *Cucumis sativus* uncharacterized P450; CsCYP88L2, P450 making cucurbitadienol C19 hydroxylation; CpCYP88L4, *Cucurbita pepo* CYP88L4 homolog; CsCYP88L4, *Cucumis sativus* uncharacterized P450; McCYP88L8, *Momordica charantia* P450 making cucurbitadienol C7 hydroxylation; McCYP88L7, *Momordica charantia* P450 making cucurbitadienol C19 and C7 hydroxylation; CpCYP88A60, *Cucurbita pepo* putative ent-kaurenoic acid hydroxylase; CsCYP88A60, *Cucumis sativus* putative ent-kaurenoic acid hydroxylase; CsCYP89A2-homologs, *Cucumis sativus* used as outgroup. Accession number of sequences are given in **Table S5**. Two Cucumis sativus sequences homologous to Arabidopsis thaliana CYP89A2 were used as an outgroup.

Enzymes performing the C19 hydroxylation on cucurbitadienol have only been characterized in cucumber and Momordica catalyzed by the P450s CYP88L2 (Shang et al., 2014) and CYP88L7 (Takase et al., 2019), respectively. Despite squash being phylogenetically closer to cucumber than Momordica, there are no reports of cucurbitacins with a C19 hydroxylation in squash. To investigate if the absence of C19 hydroxylated cucurbitacins in squash was due to a lack of a CYP88L2 functional ortholog, we collected sequences and built a phylogenetic tree with CYP88 aa sequences from Cucurbitaceae species. Through this work we identified misannotations and mislabels in genomes of the cucurbit genomic database and previous papers (**Figure S5**). The reannotation of the 24kb long Cp4.1LG15g03520 gene model of squash containing homologous sequence to *CsCYP88L2*, resolved three CsCYP88L2 paralogs termed CpCYP88L4 (Genbank: MZ695222), and the probable pseudogenes CYP88L9P and CYP88L10P (**Figure S5**). The phylogenetic analysis revealed that CYP88s from the Cucurbiteae tribe form a separate clade arising from a CsCYP88L4 ancestor in the Cucurbitaceae. In addition, CsCYP88L2 apparently evolved more recently from a CsCYP88L4 ancestor that duplicated before the split of cucumber and *Citrullus lanatus*, and then underwent several duplications in cucumber; these duplicates form a different clade than that of McCYP88L7 (**Figure 6**). Theis suggests that C19-hydroxylation of cucurbitadienol evolved independently in cucumber and Momordica by recruitment from the CYP88L subfamily.

The CpCYP88L4 ORF sequence was amplified from squash root cDNA and tested for C19-hydroxylation activity by overexpression in a yeast strain producing cucurbitadienol, however, no activity was detected as compared to yeast overexpressing cucumber *CYP88L2* and momordica *CYP88L7* (data not shown).

**Figure 7:**
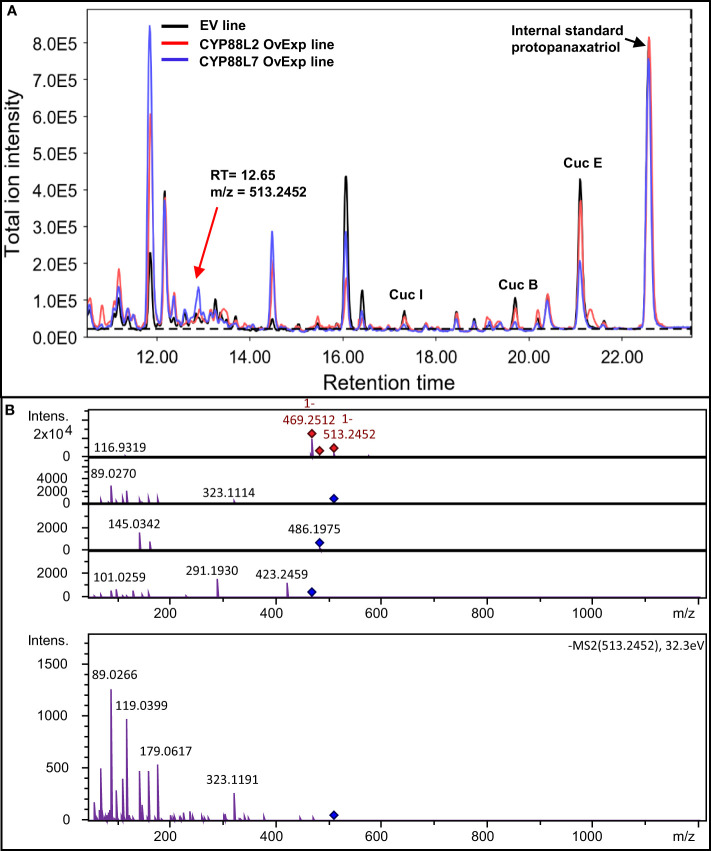
CYP88L2 and CYP88L7 appear to hydroxylate cucurbitacin intermediates in metabolically engineered *Cucurbita pepo* hairy roots. **(A)** Total ion chromatograms of HPLC-QToF analysis from *Cucurbita pepo* hairy roots empty vector, *CYP88L2* overexpression and *CYP88L7* overexpression lines. The new peak at 12,65 min is indicated with an arrow. **(B)** MS2 for the ion 513.2779 [M+COOH]^-^ mass spectra at minute 12.65 *Cucurbita pepo* overexpressing *CYP88L2* and *CYP88L7*. Red diamonds represent ions detected in MS1 destined for fragmentation, while blue diamonds are these parental ion fragmented individually in the MS2 detector.

Next, metabolic engineering of new cucurbitacins in squash HRs was attempted by overexpressing *CsCYP88L2* and *McCYP88L7*. Overexpression of either *CsCYP88L2* or *McCYP88L7* in squash HRs resulted in an obvious reduction of Cucs B,E&I compared to EV in some of the lines, however, cucurbitacins reduction was not statistically significant (**Figure S6**). No ions with m/z values corresponding to Cucs B,D,E&I with an additional hydroxyl group (**Supplementary File 2**) could be detected in the chromatograms of transgenic HRs overexpressing CsCYP88L2 or McCYP88L7. However, a thorough analysis of the LC-MS/MS chromatograms for putative cucurbitacin derived compounds revealed a peak eluding at 12.65 min in both the *CsCYP88L2* and *McCYP88L7* overexpressing lines (**Figure 7A**). The 12.65 min. peak is composed of several ions; the ion of m/z value of 469.2512 is found in both the EV and transgenic HR lines but the ion of m/z value 513.2452 [M-H]^-^ is unique to transgenic lines (**Figure S7A**). The mass of the parental ion and fragmentation pattern for this candidate peak could correspond to a triterpenoid with a molecular formulas of: C_30_H_42_O_7_ (containing four hydroxyl and three carbonyl groups as well as three double bonds), or C_32_H_50_O_5_ with two different compositions of functional groups (two hydroxyl, one carbonyl and 1 acetyl as well as two double bonds or three hydroxyl and one acetyl as well as three double bonds) (**Supplementary File 2**). Possible cucurbitacin intermediate structures predicted for these molecular formulas are illustrated in **Figures S7B-D**. Due to the low accumulation levels of the putative new cucurbitacin, it was not possible to purify enough compound for structure elucidation using NMR.

Finally, to confirm the observation that Cucs B,D,E&I could not be hydroxylated by CYP88L2 and CYP88L7, the Cucs B,D,E&I were fed to yeast cells overexpressing these genes. As a positive control, when yeast cells overexpressing CpACT1 were fed Cuc I, acetylation of Cuc I to Cuc E was effectively observed (**Figure S8**). However, when Cucs B,D,E&I were individually fed to yeast cells overexpressing CsCYP88L2 or McCYP88L7, no additional peaks were observed (**Figure S8**). These results indicated an inability of CsCYP88L2 and McCYP88L7 to hydroxylate Cucs B,D,E&I, suggesting these enzymes work earlier in the cucurbitacin pathway, and may also explain the low level of the 12.65 min. peaked observed in *CsCYP88L2* and *McCYP88L7* overexpressing HR lines.

In summary, the phylogenetic analysis of the CYP88L subfamily in the Cucurbitacea suggests that the C19 hydroxylation trait by CsCYP88L2 and McCYP88L7 likely evolved independently in cucumber and Momordica. Both P450s proved capable of creating a new peak in the LC-MS analysis, suggesting cucurbitacins with unreported combinations of functional groups is possible in squash HRs through this strategy. Albeit, determination of this peak and strategies to optimize its production would need to be pursued in later research taking into account the fact that CsCYP88L2 and McCYP88L7 act early in cucurbitacin biosynthesis, as suggested by cucurbitacin-feeding experiments in yeast.

## 4 Discussion

### 4.1 bHLH clade II TFs can be used to induce cucurbitacin production across the Cucurbitaceae

The results in this paper show that cucurbitacin biosynthesis in Cucurbitaceae species can be increased by overexpression of *CpCUCbH1* in squash HRs and by environmental conditions such as growth in TIRs. Previously the class II bHLH TFs CsBl and CsBt in cucumber and ClBr in *Citrullus lanatus* were reported to regulate cucurbitacin biosynthesis in leaf, fruit and roots, respectively (Zhou et al., 2016). In this study it was observed that the bHLH class II in the Cucurbitaceae splits into two clades in which one contains functional homologs of CsBl. In particular, CpCUCbH1 can induce biosynthesis of Cucs B,D,E&I in squash HRs, Cuc C in cucumber cotyledons but only Cuc D in *E. elaterium* leaves. Furthermore, transient overexpression of CpCUCbH1 alone did not induce cucurbitacin biosynthesis in Luffa and squash cotyledons. Consistent with the latter observation, Brzozowski et al. (Brzozowski et al., 2020) reported that cucurbitacin biosynthetic genes are not expressed in squash cotyledons and thus accumulation of cucurbitacins in cotyledons depends on transport of these metabolites from the roots; in addition, cucurbitacin accumulation trait in squash appears to be cultivar dependent.

It is known that bHLH TFs may make heterocomplexes with MYB TFs to be functional, and that the function of these heterocomplexes can be regulated by protein interactions such as by WD40 repeat proteins (Li, 2014; Pireyre and Burow, 2015). The absence of a MYB TF or presence of a WD40 protein could be a reason why cotyledons of squash and Luffa transiently overexpressing CpCUCbH1 did not produce cucurbitacins. When Shang et al. (Shang et al., 2014) restored Cuc C production in cotyledons of the non-bitter cucumber line XY-3 by transiently expressing CsBl, they were complementing a mutation in the CsBl TF, and since cucumber cotyledons typically produce Cuc C it is probable that the cofactors to induce Cuc biosynthesis were also present. In conclusion, further work is needed to understand the intricacies of cucurbitacin biosynthesis regulation throughout the Cucurbitaceae species and plant tissues.

When HRs were grown in different conditions, it was found that growth in TIR boosted Cuc production and induced the known biosynthetic genes (**Figures 3**, **4**). The work described here was consistent with other papers where culture of HRs in TIR had been shown to increase yields of plant specialized metabolites (Thakore et al., 2017; Kochan et al., 2018); but perhaps, more interesting in this study was that this increase in expression of cucurbitacin biosynthetic genes and cucurbitacin production was not related to CpCUCbH1 expression (**Figures 3**, **4**). In addition, class IV bHLH TFs inducing terpenoid production in *Medicago truncatula* and *Cataranthus roseus* are known to be induced by jasmonate signaling pathway, but this was apparently not the case for the expression of CpCUCbH1 since expression did not increase after MeJA treatment.

### 4.2 Potentially new cucurbitacin structures can be metabolically engineered in squash HRs and tabacco leaves

The cucurbitacin pathway continues to evolve in the Cucurbitaceae family, and thus not all Cucurbitaceae species have the same cucurbitacin profiles (**Figure 1**). Cucs B,D,E&I are the most widespread cucurbitacins, their presence can be observed starting in the Telfairieae tribe after the split from the Momodiceae (Rehm et al., 1957; Schaefer et al., 2009); nevertheless, cucumber in the Benincaseae tribe has lost the ability to produce Cucs B,D,E&I and instead biosynthesizes Cuc C. Cucurbitacins in the Momordiceae species in general lack the C16 hydroxylation (Okabe et al., 1982). In the case of cucumber, the absence of Cucs B,D,E&I is due to the lack of function of the CYP81Q59 catalyzing the C2 hydroxylation (**Figure 2**) (Shang et al., 2014). We identified an ACT, IaCUCA1, from *I. amara* that acetylates at the C3 postion. A phylogentic analysis showed that it evolved independently of the Cucurbitaceae ACTs that acetylate the C16 and C25 hydroxyl groups (Thakore et al., 2017), but evolved out of a clade in the Brassicaceae which has a predisposition for acetylating triterpenoids. To our knowledge, the acetylation of the hydroxyl group in C3 of cucurbitadienol has not been reported before. The fact that no 3-acetyl-16-hydroxy-cucurbitadienol was found in crude extracts of *I. amara*, most likely reflects the promiscuity of IaCUCA1; indeed, ACTs have been shown to be promiscuous acting in several substrates like the AtTHAA2 (Huang et al., 2019) from which IaCUCA1 diverged from. Nevertheless, the ability to create novel cucurbitacin modifications, reflects that *I. amara* represents an additional potential reservoir for cucurbitacin-modifying genes as the pathway has evolved independently in the two lineages.

Cucumber and Momordica diverged 46 ± 3 MY (Schaefer et al., 2009), but they both report the ability to hydroxylate at the C19 position. In contrast, there are no reports of cucurbitacins with C19 hydroxylation in squash despite of it diverging from cucumber only 30 ± 4 MY. The data presented in this paper shows that CsCYP88L2 and McCYP88L7 enzymes are responsible for the C19-hydroxylation in cucumber and Momordica, respectively, and suggests that CsCYP88L2 and McCYP88L7 must act early on the cucurbitadienol backbone as they are unable to hydroxylate highly oxygenated Cucs B,D,E&I (**Figures 6**; **S8**) possibly due to steric hindrance in the active site. Taking into consideration the suggested early action of these P450s and the lack of ions with m/z values for a deacetylated cucurbitacin in the MS2 spectra (**Figure 7B**), then a likely structure for the new observed peak could be one of those with molecular formula of C_30_H_42_O_7_ in **Figure S7B**; especially the middle structure as this molecule would not be acetylated by the promiscuous CpACT1.

## 5 Conclusions

In this paper, we show that squash HR can be used to produce cucrbitacins and that through metabolic engineering cucurbitacin structures can be modified and even novel cucrbitacin structures can be generated. We show that overexpression of specific transcription factors is an effictive strategy to boost cucurbitacin levels in squash HR grown on agar plates. This complements our earlier findings that overexpression of squalene epoxidase in HR and *Nicotiana benthamiana* increase cucurbitacin/triterpenoid levels by increasing the availability of the precursor 2,3-oxidosqualene (Almeida et al., 2018; Dong et al., 2018). In the present work we learned that the transcription factor CpCUCbH1 can induce cucurbitacins in several Cucurbitaceae species, indicating it can be used as a metabolic engineering tool in this family; however, the cucurbitacin-induction mechanism of CpCUCbH1 appears only partially conserved in *E. elaterium* in the Bryonieae tribe. In addition, convergent evolution of cucurbitacins in *I. amara* makes this plant a potential reservoir for genes that could generate novel cucucrbitacin structures with potentially new or improved bioactivities through metabolic engineering. Finally, using cucurbitacins as an example, this paper provides initial evidence that a HR platform can be used to modify and increase the production of specialized metabolites for which the biosynthetic pathway has not been fully elucidted.

## Data availability statement

The original contributions presented in the study are included in the article/**Supplementary Materials**. Further inquiries can be directed to the corresponding author.

## Author contributions

AA performed most of the experiments and analysis and drafted the article. TT was involved in the *Cucumis sativus* CYP88L2 experiments. MR was involved in the yeast experiments. LD and BK were involved in the *Iberis amara* experiments. NC-Q was involved in experimental design and construction of vectors. SK and SB supervised the work and revised the manuscript. All authors contributed to the article and approved the submitted version.

## Funding

SB, AA, and MR were supported by grants from the Independent Research Fund Denmark grant No. 7017-00275B and Novo Nordisk Foundation grant No. NNF17OC0027646.

## Acknowledgments

The authors would like to thank Bruno Trevenzoli Favero and Henrik Vit Lütkin for providing the temporary immersion reactors used in this study. In the same manner, the authors thank Yadira Peña Garcia for providing the winrhizo data. The authors would also like to thank David Nelson for assisting in the annotation and naming of P450s in this manuscript.

## Conflict of interest

The authors declare that this study received funding from the Novo Nordic Foundation. The funder was not involved in the study design, collection, analysis, interpretation of data, the writing of this article, or the decision to submit it for publication.

## Publisher’s note

All claims expressed in this article are solely those of the authors and do not necessarily represent those of their affiliated organizations, or those of the publisher, the editors and the reviewers. Any product that may be evaluated in this article, or claim that may be made by its manufacturer, is not guaranteed or endorsed by the publisher.

## Supplementary material

Supplementary Figure 1
*Cucurbita pepo* has a higher transformation efficiency for hairy root line production than *Iberis umbellata*. Stereomicroscope images of *Cucurbita pepo* hairy roots overexpressing *mRFP*, on the left are the bright-field images **(A, C)** and on the right is the fluorescence through a 590 LongPass emission filter **(B, D)**. Stereomicroscope images of *Iberis umbellata* hairy roots overexpressing mRFP on the left are the bright-field images **(E, G, I)** and on the right is the fluorescence through a 590 LongPass emission filter **(F, H, J)**. **(K)** Example of hairy root lines generated from one transformation round. **(L)** Different tissues from *Iberis umbellata* transformed for hairy root production. The table below list the transformation frequencies of *C. pepo* versus *I. amara.*

Supplementary Figure 2 Temporary immersion reactors reduce within line variation of *Cucurbita pepo* hairy roots. Representative hairy root showing variation on growth in 200 ml Erlen Meyer flasks **(A-C)**. **(D)** Fresh weight yields of different hairy root lines transformed with different vectors, either EV or overexpression of CpACT, showing more consistent growth in TIR. Pictures of hairy roots grown in temporary immersion reactors **(E, F)**. Data points represent means with standard deviation (*n* = 6), (*p* < 0.05)

Supplementary Figure 3 Higher accumulation of cucurbitacins by overexpression of *CpCUCbH1* results in hairy roots with less branching and reduced total length. Representative hairy root lines overexpressing *CpCUCbH1* with three technical replications within each plate **(A-C)**. Representative empty vector hairy root lines with technical replicates within each plate **(D-F)**. Three root phenotypes: Primary root length **(G)**, Number of root tips **(H)** and Total root length **(I)** assessed by WinRhizo for empty vector and CpCUCbH1 overexpressing hairy roots

Supplementary Figure 4 Overexpression of *CpCUCbH1* alone does not induce *de novo* cucurbitacin biosynthesis in tissues that do not already accumulate cucurbitacins. Representative confocal image of *Cucurbita pepo* **(A)**, *Cucumis sativus* **(B)**, *Luffa cylindrical* **(C)** cotyledons and *Ecballium elaterium* leaves **(D)** overexpressing GFP to assess successful transformation. Confocal laser scanning microscopy images were collected 5 days after agroinfiltration of cotyledons/leaves. **(E)** Total ion chromatograms of UHPLC-QToF analysis from infiltrated cotyledons of *Luffa cylindrica* showing absence of cucurbitacins. **(F)** Total ion chromatograms of UHPLC-QToF analysis from infiltrated cotyledons of *Cucurbita pepo* showing absence of cucurbitacins.

Supplementary Figure 5 Tandem duplications of the CYP88L subfamily in Cucurbitaceae species have resulted in expansion of CYP88L paralogs. **(A)** Alignment of CYP88 amino acid sequences showing 27 missing amino acids in the sequences misannotated on available databases (Cucurbit Genomics Data Base (Csa6G088690) and NCBI (NP_001295866)). The *Cs*C*YP88L2* sequence obtained from cucumber leaf cDNA in this study (Genbank accession: MZ695220) encoded 27 aa more at the C-terminal region than sequences deposited in CuGenDB and NCBI. Multiple-pairwise aligments showed that the previously reported *CYP88L2* sequence had only 463 aa while all other CYP88s were 489-490 aa long, indicating it had been misannotated. **(B)** Genome browser image from Cucurbit Genomics Database displaying the chromosomal region of *Cucurbita pepo* where CYP88L paralog sequences are localized; the image shows that the region is misannotated as a single gene. The line at the left of the predicted transcript represents the start site (5’-end) while the arrow at the right represents the end (3’-end). **(C)** Graphical representation of chromosal region in *Cucurbita pepo* containing CYP88L sequences after reannotation with FgenesH software; this representation shows the region in Figure S6B actually contains 12 open reading frames out of which 3 are CYP88L paralogs. **(D)** Graphical representation of chromosomal region in *Cucurbita moschata* containing CYP88L sequences after reannotation with FgenesH software. **(E)** Graphical representation of chromosomaal region in *Cucurbita maxima* containing CYP88L sequences after reannotation with FgenesH software. **(F)** Graphical representation of chromosomal region in *Citrullus lanatus* containing CYP88L sequences after reannotation with FgenesH software. **(G)** Graphical representation of chromosomal region in *Cucumis sativus* containing CYP88L sequences after reannotation with FgenesH software.

Supplementary Figure 6 Overexpression of *CsCYP88L2* and *McCYP88L7* does not significantly lower levels of cucurbitacins in *Cucurbita pepo* hairy roots. Comparison of Cucurbitacin I **(A)**, B **(B)** and E **(C)** between *C. pepo* hairy root lines overexpressing different gene constructs shows there is no significant drop in the cucurbitacin levels compared to the EV lines. The same constructs were tested 2 and 3 months after transformation. Data points represent means with standard deviation (*n* = 6), (*p* < 0.05).

Supplementary Figure 7 The m/z ion 513.2452 is unique to transgenic hairy roots of squash overexpressing either *CYP88L2* or *CYP88L7*. **(A)** Extracted ion chromatogram (m/z= 513-513.5) for UHPLC-QToF analysis (negative mode) for EV and *CYP88L2* or *CYP88L7* overexpressing hairy roots. **(B)** Possible cucurbitacin structures for an ion of m/z= 513-513.5 with molecular formula C_30_H_42_O_7_ containing four hydroxyl and three carbonyl groups as well as three double bonds. **(C)** Possible cucurbitacin structures for an ion of m/z= 513-513.5 with molecular formula C_32_H_50_O_5_ containing two hydroxyl, one carbonyl and 1 acetyl groups as well as two double bonds. **(D)** Possible cucurbitacin structures for an ion of m/z= 513-513.5 with molecular formula C_32_H_50_O_5_ containing three hydroxyl and one acetyl groups as well as three double bonds.

Supplementary Figure 8 McCYP88L7 acts early on in the cucurbitacin pathway as it does not act on highly oxygenated cucurbitacins like Cucs E&I. **(A)** Extracted ion chromatograms (m/z= 532-533; 574-575) for UHPLC-QToF runs (positive mode) of yeast cultures overexpressing Mc*CYP88L7* supplemented with Cucs E&I. **(B)** Extracted ion chromatograms (m/z= 559-560; 601-602) for UHPLC-QToF runs (negative mode) of yeast cultures overexpressing *CpACT* fed Cuc I to generate Cuc E to prove that supplementing with cucurbitacins to yeast is possible.

Supplementary Table 1 List of primers used in this study.

Supplementary Table 2 List of plasmids used for yeast experiments.

Supplementary Table 3 List of seed providers for Cucurbitaceae plant species used in this study.

Supplementary Table 4 List of yeast strains used and constructed in this study.

Supplementary Table 5 Details of sequences used for sequence alignments in **Figure 2**, **Figure 4** and **Figure 5**.

Supplementary File 1 Structure elucidation of 3-O-acetyl-16β-hydroxycucurbitadienol using NMR.

Supplementary File 2 Calculated masses of potential intermediates in the biosynthesis of cucurbitacins.
