# Supplementary material for: Metabolic engineering of cucurbitacins in Cucurbita pepo hairy roots
Source: Front Plant Sci. 2022 Dec 5;13:1021907. doi: 10.3389/fpls.2022.1021907 (PMC9760960; doi:10.3389/fpls.2022.1021907)
Supplement: Supplementary Figure 1 — Cucurbita pepo has a higher transformation efficiency for hairy root line production than Iberis umbellata. Stereomicroscope images of Cucurbita pepo hairy roots overexpressing mRFP, on the left are the bright-field images (A, C) and on the right is the fluorescence through a 590 LongPass emission filter (B, D). Stereomicroscope images of Iberis umbellata hairy roots overexpressing mRFP on the left are the bright-field images (E, G, I) and on the right is the fluorescence through a 590 LongPass emission filter (F, H, J). (K) Example of hairy root lines generated from one transformation round. (L) Different tissues from Iberis umbellata transformed for hairy root production. The table below list the transformation frequencies of C. pepo versus I. amara. [file DataSheet_1.pdf]

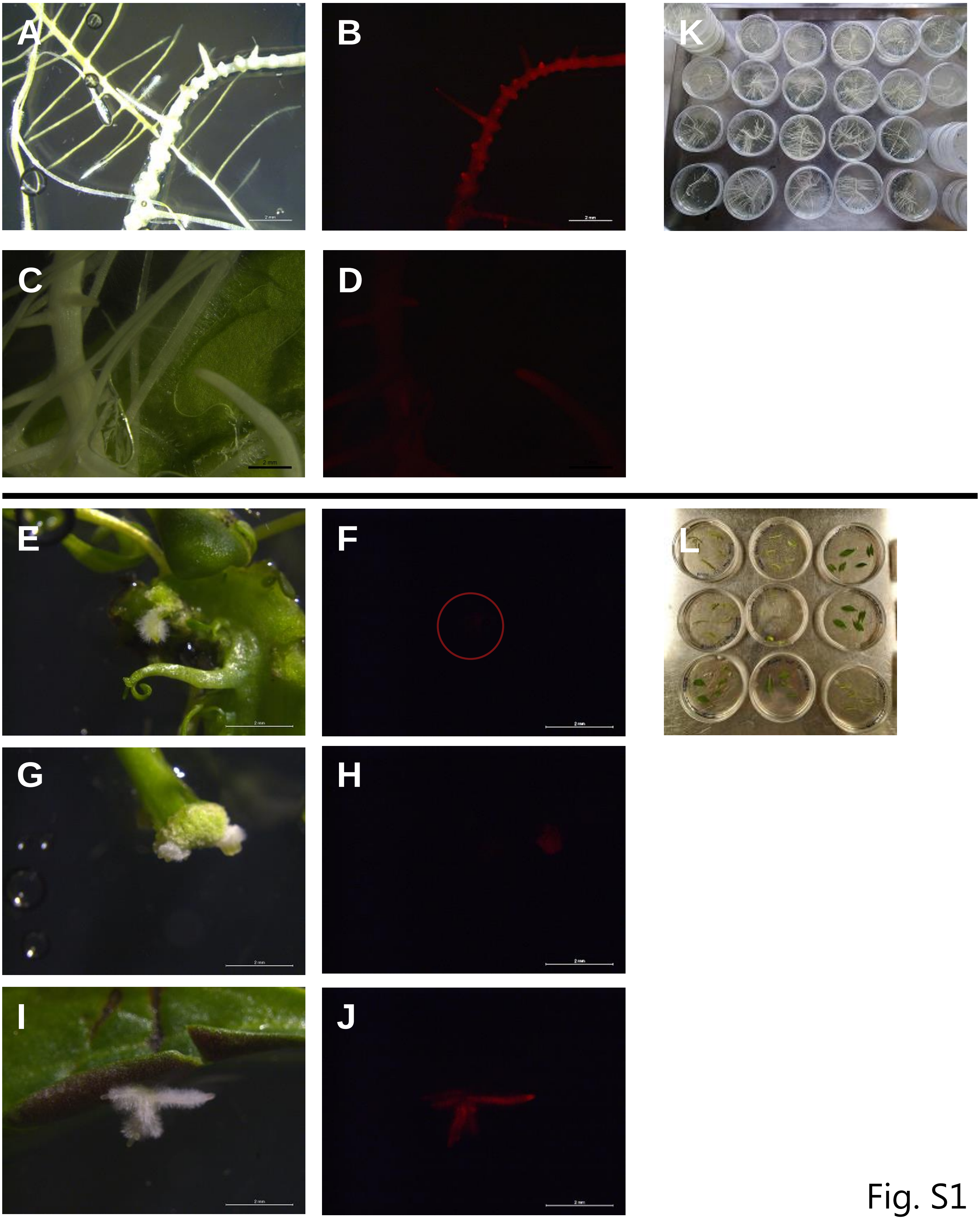

Fig. S1

Species	Tissue	Number	Transgenic hairy roots generated	Transformation efficiency
Iberis umbellata	Stem	26	2	7,69% (succeeded once)
Iberis umbellata	Leaf	16	1	6,25% (succeeded once)
Cucurbita pepo	Cotyledon	20	14±4,32	46,66±14,40%

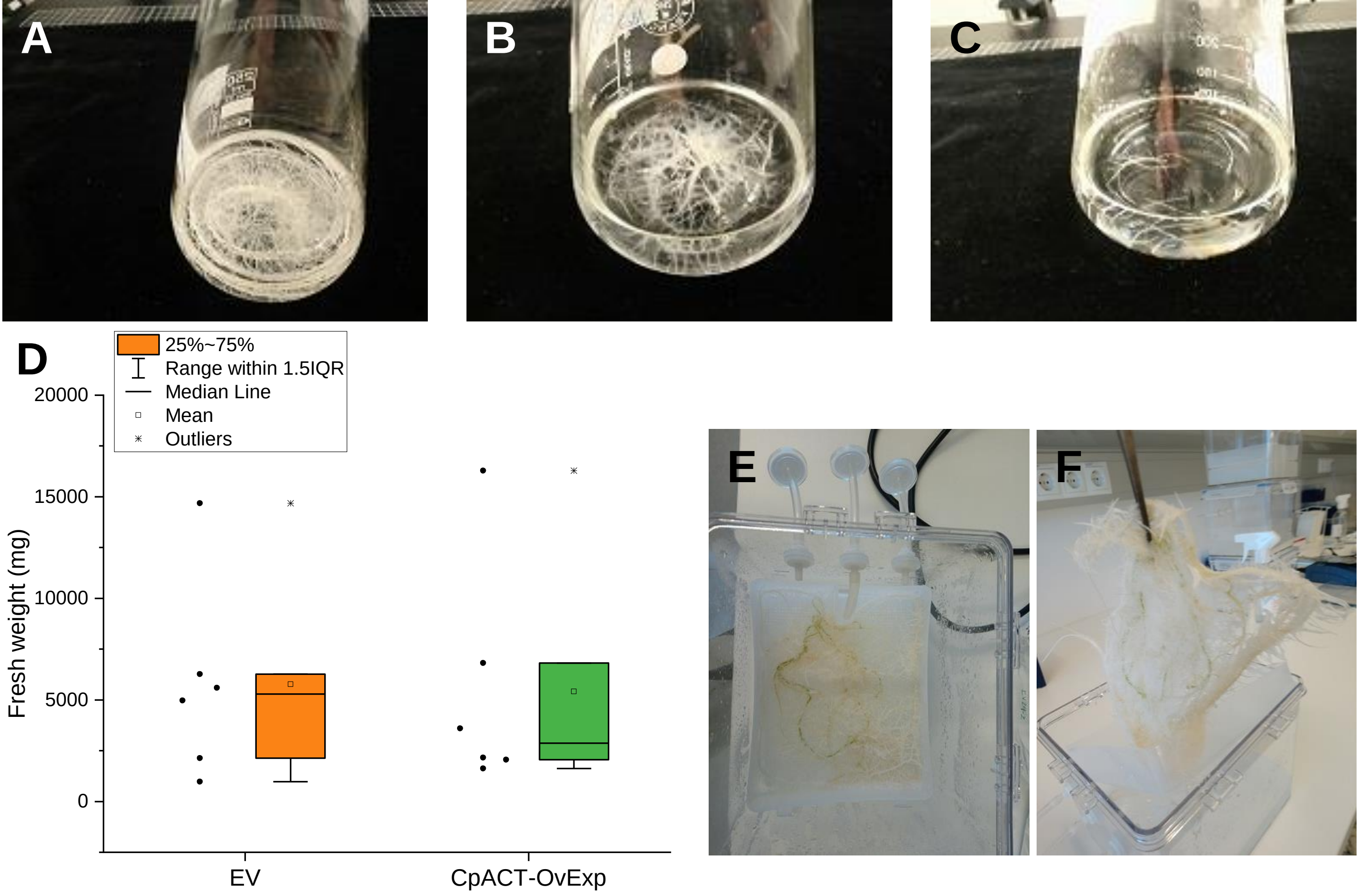

Construct	Initial fresh weight (mg)	Final Fresh weight (mg)	Weight gain fold	Final dry weight (%)
EV	848.98±41.68	3990±2306	4.78±2.75	9.76±1.6%
CpACT-OvExp	Not measured	3248±2129	Not measured	10.42+2.01%

Fig. S2

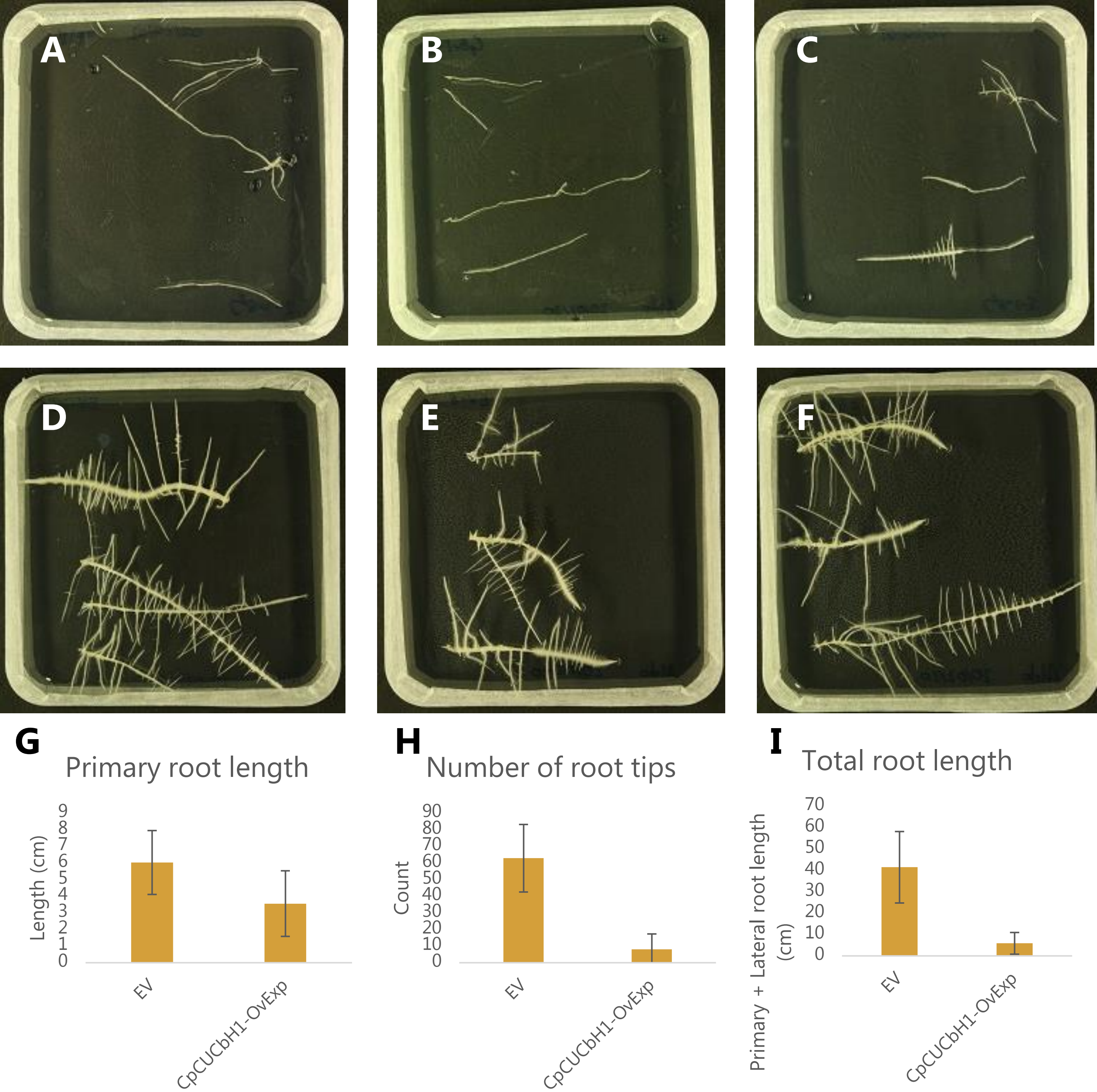

A
B
C
D
E
F
G
Primary root length
Length (cm)
9
8
7
6
5
4
3
2
1
0
EV
CpCUCbH1-OvExp
H
Number of root tips
Count
90
80
70
60
50
40
30
20
10
0
EV
CpCUCbH1-OvExp
I
Total root length
Primary + Lateral root length (cm)
70
60
50
40
30
20
10
0
EV
CpCUCbH1-OvExp

Fig. S3

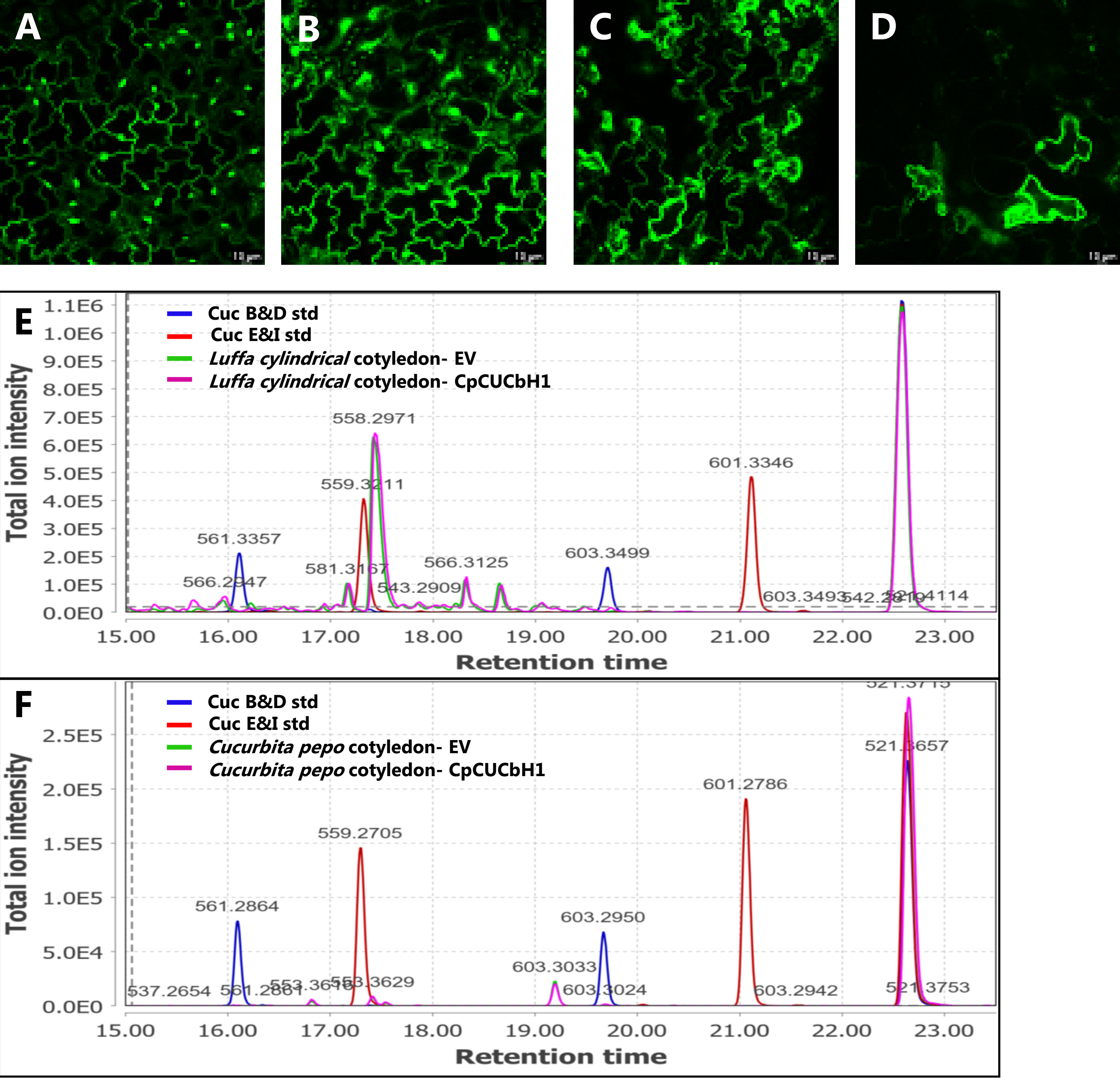

A
B
C
D
E
Cuc B&D std
Cuc E&I std
Luffa cylindrical cotyledon- EV
Luffa cylindrical cotyledon- CpCUCbH1
Total ion intensity
Retention time
F
Cuc B&D std
Cuc E&I std
Cucurbita pepo cotyledon- EV
Cucurbita pepo cotyledon- CpCUCbH1
Total ion intensity
Retention time

Fig. S4

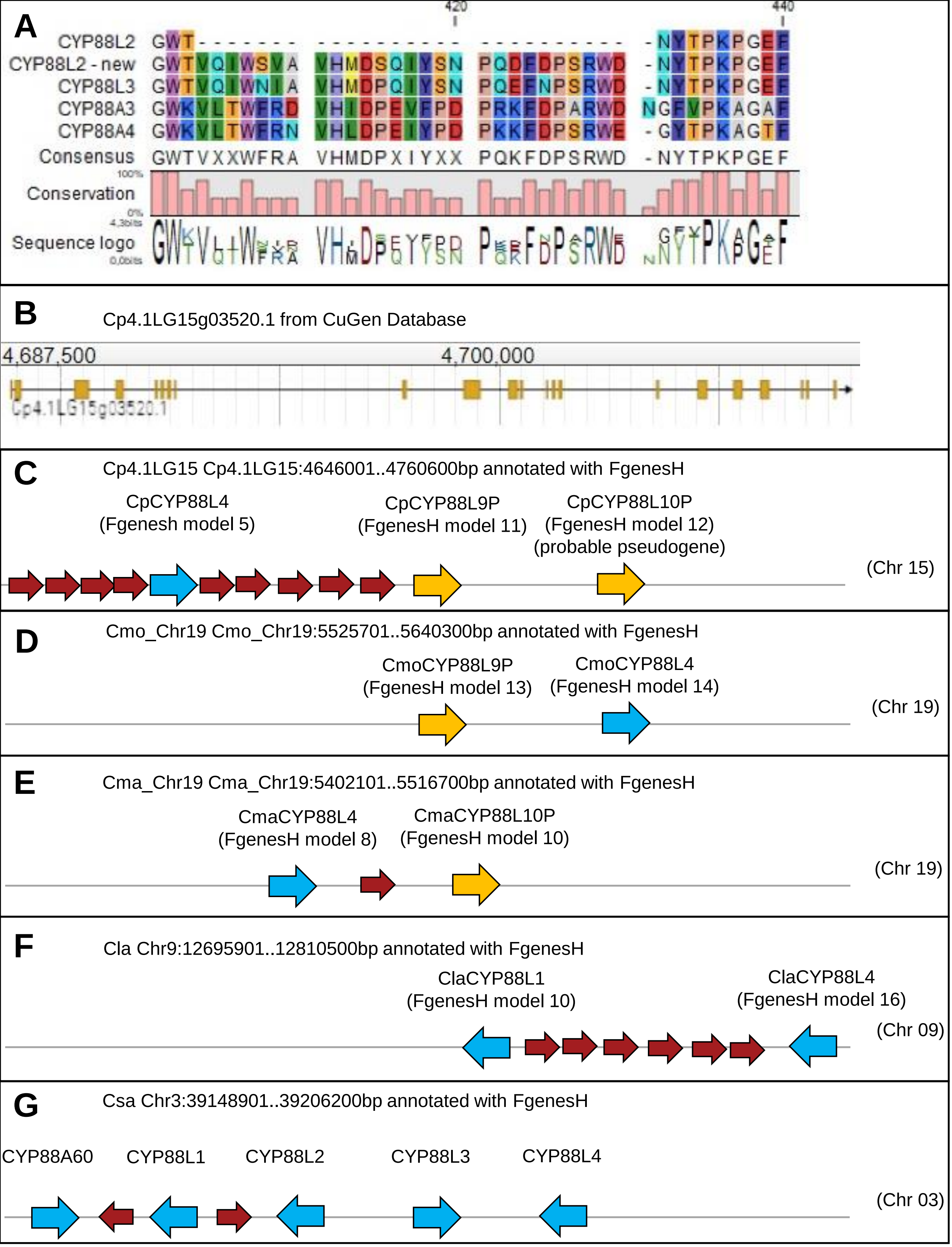
A
CYP88L2
CYP88L2 - new
CYP88L3
CYP88A3
CYP88A4
Consensus
GWTVXXWFRA VHMDPXIYXX PQKFDPSRWD -NYTPKPGEF
Conservation
Sequence logo
B
Cp4.1LG15g03520.1 from CuGen Database
4,687,500
4,700,000
C
Cp4.1LG15 Cp4.1LG15:4646001..4760600bp annotated with FgenesH
CpCYP88L4
(Fgenesh model 5)
CpCYP88L9P
(FgenesH model 11)
CpCYP88L10P
(FgenesH model 12)
(probable pseudogene)
(Chr 15)
D
Cmo_Chr19 Cmo_Chr19:5525701..5640300bp annotated with FgenesH
CmoCYP88L9P
(FgenesH model 13)
CmoCYP88L4
(FgenesH model 14)
(Chr 19)
E
Cma_Chr19 Cma_Chr19:5402101..5516700bp annotated with FgenesH
CmaCYP88L4
(FgenesH model 8)
CmaCYP88L10P
(FgenesH model 10)
(Chr 19)
F
Cla Chr9:12695901..12810500bp annotated with FgenesH
ClaCYP88L1
(FgenesH model 10)
ClaCYP88L4
(FgenesH model 16)
(Chr 09)
G
Csa Chr3:39148901..39206200bp annotated with FgenesH
CYP88A60
CYP88L1
CYP88L2
CYP88L3
CYP88L4
(Chr 03)

Fig. S5

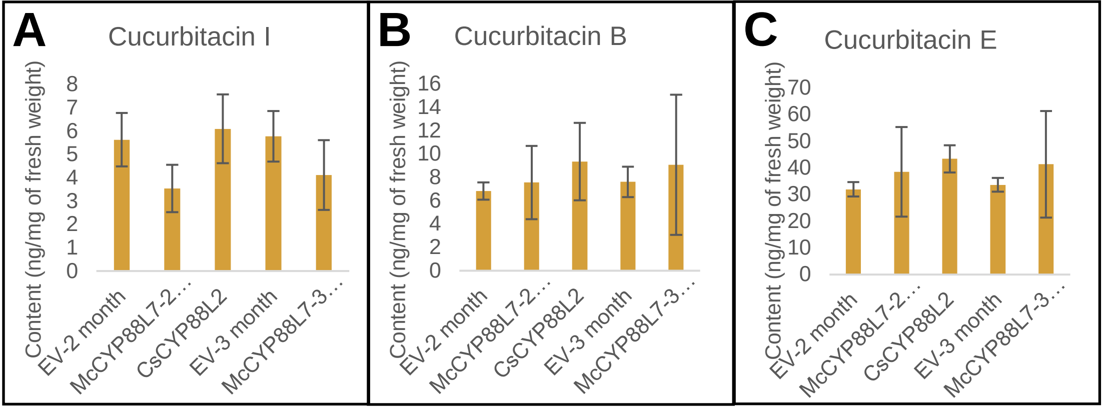

A
Cucurbitacin I
Content (ng/mg of fresh weight)
0
1
2
3
4
5
6
7
8
EV-2 month
McCYP88L7-2…
CsCYP88L2
EV-3 month
McCYP88L7-3…
B
Cucurbitacin B
Content (ng/mg of fresh weight)
0
2
4
6
8
10
12
14
16
EV-2 month
McCYP88L7-2…
CsCYP88L2
EV-3 month
McCYP88L7-3…
C
Cucurbitacin E
Content (ng/mg of fresh weight)
0
10
20
30
40
50
60
70
EV-2 month
McCYP88L7-2…
CsCYP88L2
EV-3 month
McCYP88L7-3…

Fig. S6

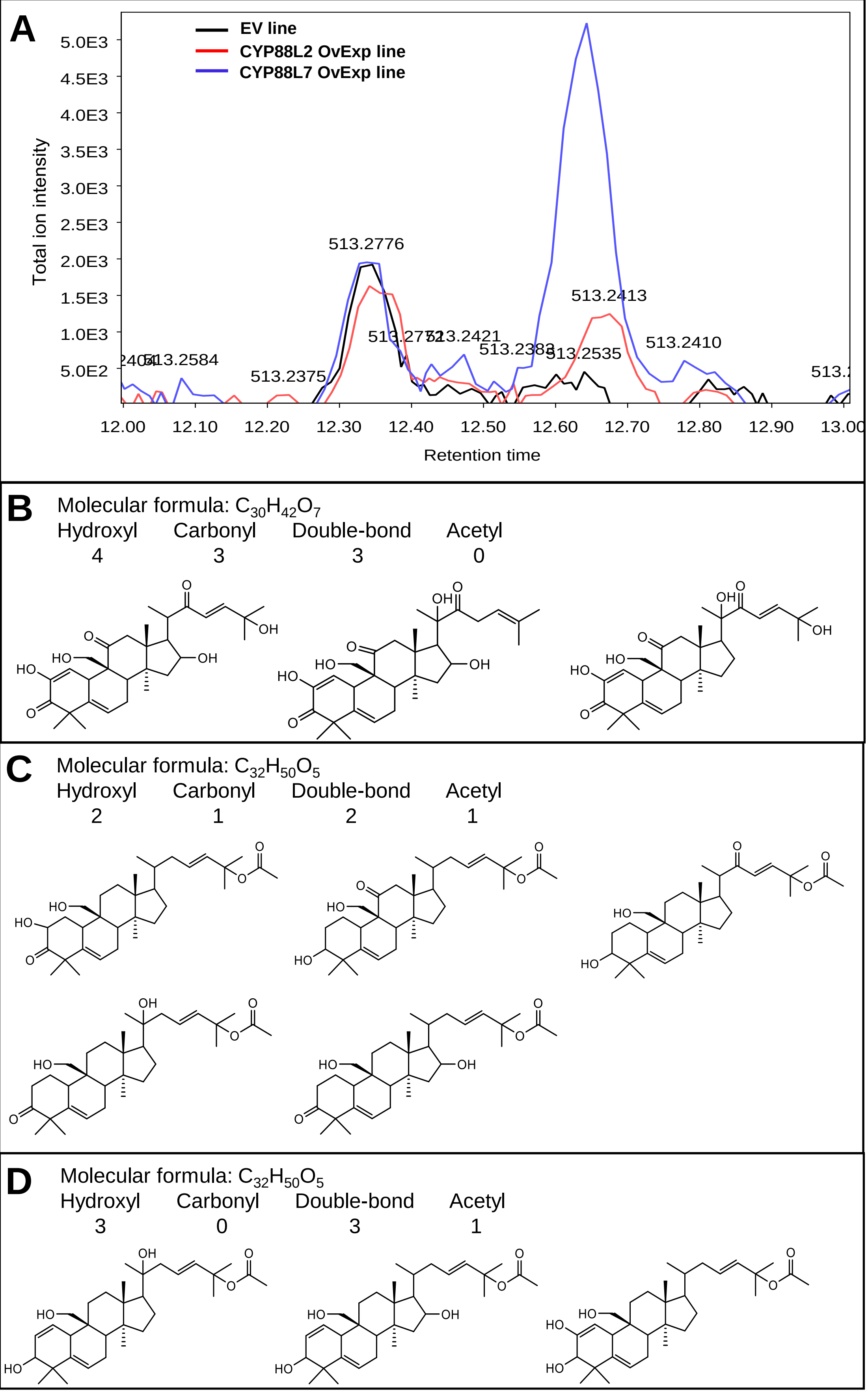
A
EV line
CYP88L2 OvExp line
CYP88L7 OvExp line
Total ion intensity
5.0E3
4.5E3
4.0E3
3.5E3
3.0E3
2.5E3
2.0E3
1.5E3
1.0E3
5.0E2
513.2776
513.2413
513.2421
513.2383
513.2535
513.2410
513.2584
513.2375
12.00
12.10
12.20
12.30
12.40
12.50
12.60
12.70
12.80
12.90
13.00
Retention time
B
Molecular formula: $C_{30}H_{42}O_7$
Hydroxyl 4
Carbonyl 3
Double-bond 3
Acetyl 0
C
Molecular formula: $C_{32}H_{50}O_5$
Hydroxyl 2
Carbonyl 1
Double-bond 2
Acetyl 1
D
Molecular formula: $C_{32}H_{50}O_5$
Hydroxyl 3
Carbonyl 0
Double-bond 3
Acetyl 1

Fig. S7

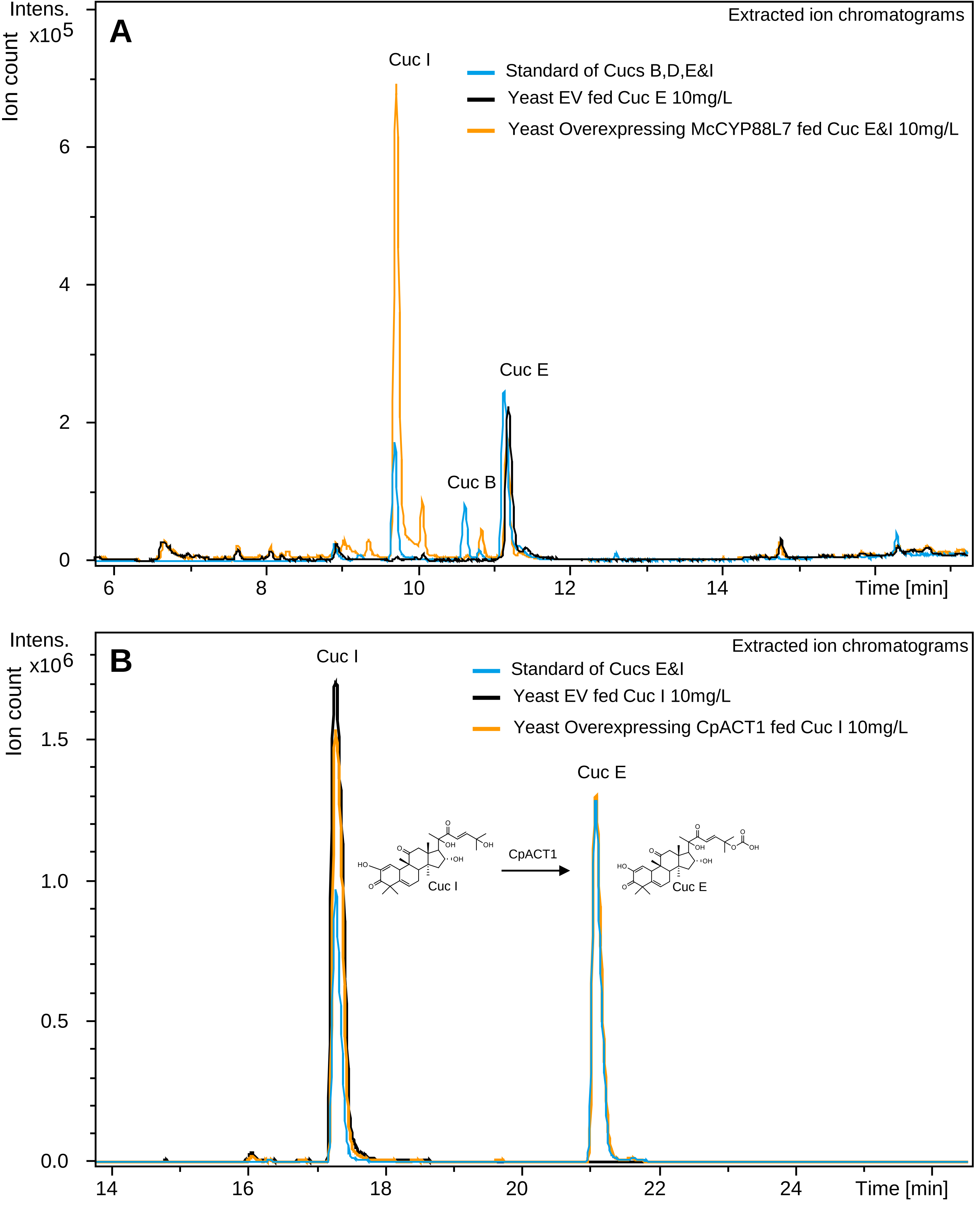

A
Intens. x10^5
Ion count
Extracted ion chromatograms
Standard of Cucs B,D,E&I
Yeast EV fed Cuc E 10mg/L
Yeast Overexpressing McCYP88L7 fed Cuc E&I 10mg/L
Cuc I
Cuc B
Cuc E
0
2
4
6
6
8
10
12
14
Time [min]
B
Intens. x10^6
Ion count
Extracted ion chromatograms
Standard of Cucs E&I
Yeast EV fed Cuc I 10mg/L
Yeast Overexpressing CpACT1 fed Cuc I 10mg/L
Cuc I
Cuc E
CpACT1
Cuc I
Cuc E
0.0
0.5
1.0
1.5
14
16
18
20
22
24
Time [min]

Fig. S8
