# Supplementary material for: Metabolic engineering of cucurbitacins in Cucurbita pepo hairy roots
Source: Front Plant Sci. 2022 Dec 5;13:1021907. doi: 10.3389/fpls.2022.1021907 (PMC9760960; doi:10.3389/fpls.2022.1021907)
Supplement: Supplementary Figure 5 — Tandem duplications of the CYP88L subfamily in Cucurbitaceae species have resulted in expansion of CYP88L paralogs. (A) Alignment of CYP88 amino acid sequences showing 27 missing amino acids in the sequences misannotated on available databases (Cucurbit Genomics Data Base (Csa6G088690) and NCBI (NP_001295866)). The CsCYP88L2 sequence obtained from cucumber leaf cDNA in this study (Genbank accession: MZ695220) encoded 27 aa more at the C-terminal region than sequences deposited in CuGenDB and NCBI. Multiple-pairwise aligments showed that the previously reported CYP88L2 sequence had only 463 aa while all other CYP88s were 489-490 aa long, indicating it had been misannotated. (B) Genome browser image from Cucurbit Genomics Database displaying the chromosomal region of Cucurbita pepo where CYP88L paralog sequences are localized; the image shows that the region is misannotated as a single gene. The line at the left of the predicted transcript represents the start site (5’-end) while the arrow at the right represents the end (3’-end). (C) Graphical representation of chromosal region in Cucurbita pepo containing CYP88L sequences after reannotation with FgenesH software; this representation shows the region in Figure S6B actually contains 12 open reading frames out of which 3 are CYP88L paralogs. (D) Graphical representation of chromosomal region in Cucurbita moschata containing CYP88L sequences after reannotation with FgenesH software. (E) Graphical representation of chromosomaal region in Cucurbita maxima containing CYP88L sequences after reannotation with FgenesH software. (F) Graphical representation of chromosomal region in Citrullus lanatus containing CYP88L sequences after reannotation with FgenesH software. (G) Graphical representation of chromosomal region in Cucumis sativus containing CYP88L sequences after reannotation with FgenesH software. [file DataSheet_2.pdf]

Supplementary file 1

Structure elucidation of *3-O-acetyl-16β-hydroxycucurbitadienol*

Isolation

For isolation of *3-O-acetyl-16β-hydroxy-cucurbitadienol*, 100 transiently-transformed-tabaco leaves co-expressing *IaCPQ* with *CYP708A16* and *IaCUCA1*, were ground in liquid nitrogen and extracted. In general, 2 g of the ground leaf powder was extracted with 50 ml of ethyl acetate, incubated at 37 °C for 1h, centrifuged for 20 min at 4000 g, and all the supernatant was transferred to a new vial and evaporated under vacuum. The pellet was suspended in 4 ml methanol. 90 µl of the methanol suspensions from both extracts were separated using a Dionex UltiMate 3000 semi-preparative high-performance liquid chromatography (Thermo Fisher Scientific, Waltham, MA, USA) equipped with a Supelco C18 column (150 x 4.0 mm i.d., 5 µm particle size), an ultraviolet-visible detector, and an automated fraction collector. The mobile phases were water (A) and acetonitrile (B). The gradient program was: 0-1 min, isocratic 50% B; 1-15 min, linear gradient 50%-100% B; 15-25 min isocratic 100% B; 25-26 min with a flow rate of 1.5 ml/min. Fractions for 3-O-acetyl-16β-hydroxy-cucurbitadienol were collected at intervals of 21.95-22.45 min, about 1 mg was collected. Purified 3-O-acetyl-16β-hydroxy-cucurbitadienol was reanalyzed using GC-MS as described above to confirm the purity of the compound.

Nuclear Magnetic Resonance (NMR) spectroscopy

NMR data were recorded at 300K using AVANCE III 600 MHz NMR spectrometers (Bruker Biospin, Rheinstetten, Germany) operating at frequency of 600.13 MHz and equipped with a 5 mm inverse probe (BBI) with z-gradient and automated tuning and matching and cooling unit BCU-05 or BCU-I, an automated sample changer (Bruker SampleJet) with sample cooling, and pre-heating station. Pulse programs for 1D and 2D NMR experiments were acquired from Bruker User Library for Small Molecular Experiments.

Conditions of 1D and 2D NMR experiments

Experiment*	PULPROG	FIDS	Size of each FID	Spectral width (ppm)	Acquisition time in sec (AQ)	Relaxation delay (D1) in sec
1D ^{1}H	*zgcppr*	512	65536	20	2.72	1.5
COSY	*cosyphpr*	64	8192 (F2)<br>256 (F1)	10	0.68 (F2)<br>0.02 (F1)	1.5
TOCSY	*mlevphpr*	80	8192 (F2)<br>256 (F1)	10	0.68 (F2)<br>0.02 (F1)	1.5
HSQC	*hsqcphpr*	512	8192 (F2)<br>256 (F1)	10 (F2)<br>240 (F1)	0.133 (F2)<br>0.007 (F1)	1.5
HMBC	*hmbcetgpl3nd*	64	4096 (F2)<br>512 (F1)	10 (F2)<br>240 (F1)	0.227 (F2)<br>0.007 (F1)	1.5

*1D ^{1}H (One-dimensional proton NMR spectroscopy); Correlation Spectroscopy (COSY); Total Correlation Spectroscopy (TOCSY); Heteronuclear Single Quantum Coherence (HSQC), Heteronuclear Multiple Bond Correlation (HMBC), NOESY (Nuclear Overhauser Effect Spectroscopy), ROESY (Rotating-frame Overhauser SpectroscoPY)

Structure elucidation of 3-O-acetyl-16β-hydroxycucurbitadienol was based on 1D and 2D NMR experiments. Approximately 1 mg of sample was dissolved in 650 μL methanol-d4. The acquired data were calibrated according to the residual solvent (methanol-d4) peaks at 3.31 ppm for ^{1}H spectra, and 49.01 ppm for ^{13}C spectra. Both 1D ^{1}H NMR spectrum and 2D NMR data were compared with the data published on cucurbitadienol and 16,22-dihydroxycucurbitadienol [1, 2]. The ^{1}H NMR spectrum depicted seven singlets corresponding to methyl groups at δ 0.85 (H30), 0.90 (H18), 0.93 (H19), 1.04 (H29), 1.07 (H28), 1.61 (H27), and 1.66 (H26). A methyl of H21 was doublet (d, δ 0.97, 6.7 Hz) due to the neighbouring proton H20 (m, δ 1.88). A doublet of triplet at δ 5.55 corresponded to the olefinic proton H6 (1.8 Hz, 6.0 Hz), which correlates to δ 120.3 in HSQC corresponding to C6. A triplet of triplet at δ 5.15 corresponded to the olefinic proton H24 (7.14 Hz, 1.36 Hz), which correlates to C24 (δ 126.3) in HSQC. The tt splitting pattern of H24 comes from coupling of two chemically not equivalent protons of H23 (AB system), with the coupling constant of 7.14 Hz, and further split into two doublets by the two methyl groups at C26 and C27, with the same coupling constant of 1.36 Hz. A hydroxylated carbon C3 (δ 80.1) showed a cross correlation with a triplet at δ 4.69, corresponding to H3 (2.7 Hz) in HSQC. Significant shift of H3 and C3 signals towards low field was related to the acetylation of the hydroxyl group at C3. The methyl group of the acetyl moiety ($C\boldsymbol{H}_3CO$, s, 1.99) was identified as singlet at δ 1.99 which depicted cross correlations with δ 20.7 ($\boldsymbol{C}H_3CO$) and δ 173.0 ($CH_3\boldsymbol{C}O$) of in HSQC and HMBC spectra, respectively. The other hydroxylated carbon C16 (δ 72.7) correlates to a signal at δ 4.38 (td, H16, 7.6, 5.8, 1.9 Hz) in HSQC. The td multiplicity of H16 is derived from three neighbouring protons, α- and β-positioned H15 (δ 1.21, δ 1.93, m) and H17 (δ 1.66, dd, 11.6, 7.6). Two protons of H15 experience different chemical environment due to the rigid pentacylic ring, thus experience different coupling to H16 and form a doublet of doublet (dd). Subsequently the H17 further splits this dd with coupling constant of 7.6 Hz and forms td. The coupling constants of 5.8 and 1.9 Hz of H16 originate from the two protons H15. The β-position of H16 in 16β,22-dihydroxycucurbitadienol has been shown through ROESY spectrum in the previous study [2], thus compound 1, which is also obtained through the activity of the same enzyme, also has H16 in β-position. In a similar manner the COSY and TOCSY spin system identification matched that of the previously reported 16β,22-dihydroxycucurbitadienol [2].

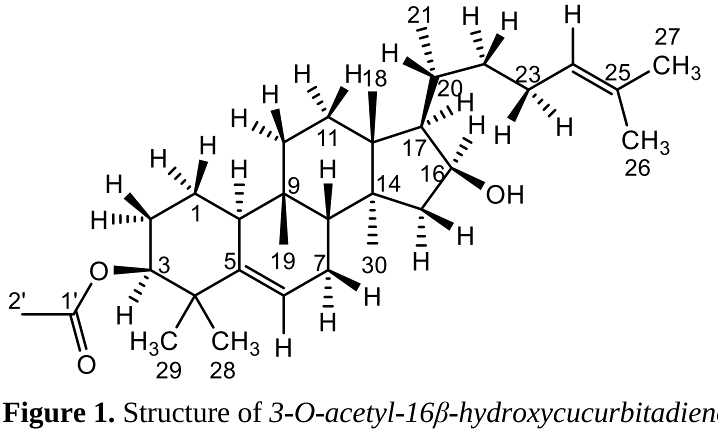

Figure 1. Structure of *3-O-acetyl-16β-hydroxycucurbitadienol..*

Table 2. One-dimensional proton and two-dimensional Heteronuclear Single Quantum Coherence (HSQC), Heteronuclear Multiple Bond Correlation (HMBC) data recorded on *3-O-acetyl-16-hydroxycucurbitadienol*. 1D ^{1}H column illustrates chemical shifts (ppm), multiplicity (s=singlet, d=doublet, t=triplet, br d=broad doublet, ddd=doublet of doublet of doublets, tt= triplet of triplet) as well as the spin-spin coupling constants (in Hz). HSQC data represents chemical shifts of protonated ^{13}C atoms while HMBC data shows correlation of proton with neighbouring ^{13}C atoms. COSY and TOCSY spectral data illustrate correlations of protons at neighbouring carbons (data not shown).

Pos.	δ ^{13}C	δ ^{1}H (nH, multiplicity, *J* in Hz)[b]	HMBC[c]
1	22.6	1.46 (1H, m)<br>1.61 (1H, m)	C-10
2	27.3	1.75 (1H, m)<br>1.90 (1H, m)	
3	80.1	4.69 (1H, t, 2.7)	
4	40.4		
5	142.6		
6	120.5	5.55 (1H, dt, 1.8, 6.0)	C-4, C-7, C-8, C-10
7	25.0	1.84 (1H, m)<br>2.41 (1H, m)	C-5, C-9
8	44.5	1.85 (1H, d, 7.5)	C-6, C-7, C-9, C-10, C-14, C-19, C-30
9	47.3		
10	39.3	2.33 (1H, m)	C-2, C-28
11	32.7	1.51 (1H, m)<br>1.69 (1H, m)	C-9, C-13, C-14
12	31.3	1.55 (1H, m)<br>1.73 (1H, m)	C-9, C-13, C-14
13	48.1*		
14	48.1*		
15	47.9	1.93 (1H m)<br>1.21 (1H, m)	C-9, C-13, C-14, C-16, C-17, C-30
16	72.7	4.38 (1H, ddd, 7.6, 5.8, 1.9)	C-15
17	55.6	1.66 (1H, dd, 11.6, 7.6)	C-9, C-20
18	14.1	0.90 (3H, s)	C-9, C-13, C-14, C-17, C-20
19	28.0	0.93 (3H, s)	C-8, C-10, C-11
20	31.1	1.88 (1H, m)	C-17, C-18
21	18.5	0.96 (3H, d, 6.7)<br>0.97	C-17, C-22
22	37.4	1.04 (1H, m)<br>1.67 (1H, m)	C-17, C-18, C-24
23	25.9	1.94 (1H, m)<br>2.08 (1H, m)	C-20, C-22, C-24
24	126.3	5.15 (1H, tt, 7.14, 1.36)	C-26, C-27
25	133.1*		
26	25.5	1.66 (3H, s)	C-24, C-25, C-27

27	17.4	1.61 (3H, s)	C-24, C-25, C-26
28	27.7	1.07 (3H, s)	C-3, C-5, C-9, C-13, C-14, C-29
29	25.1	1.04 (3H, s)	C-3, C-5, C-9, C-13, C-14, C-28
30	19.3	0.85 (3H, s)	C-8, C-9, C-13, C-14
1’	173		
2’	20.7	1.99	C1’

* Chemical shift (ppm), multiplicity and spin-spin coupling constants (Hz) of protons were obtained from the 1D ^{1}H NMR spectrum. A HSQC data enabled determination of chemical shifts of protonated carbon (^{13}C) atoms while HMBC allowed identification of carbon atoms without neighbouring protons.

Reference List

1. Shang, Y., et al., *Biosynthesis, regulation, and domestication of bitterness in cucumber.* Science, 2014. **346**(6213): p. 1084.
2. Dong, L., et al., *An Independent Evolutionary Origin for Insect Deterrent Cucurbitacins in Iberis amara.* Molecular Biology and Evolution, 2021. **38**(11): p. 4659-4673.
