# Supplementary material for: Metabolic engineering of cucurbitacins in Cucurbita pepo hairy roots
Source: Front Plant Sci. 2022 Dec 5;13:1021907. doi: 10.3389/fpls.2022.1021907 (PMC9760960; doi:10.3389/fpls.2022.1021907)
Supplement: Supplementary File 1 — Structure elucidation of 3-O-acetyl-16β-hydroxycucurbitadienol using NMR. [file DataSheet_3.pdf]

Supplementary file 2

Calculated masses of potential intermediates in the biosynthesis of cucurbitacins. The first intermediate listed is cucurbitadienol (Formula: $C_{30}H_{50}O$), precursor for all the Cucs. The rest of the potential intermediates is derived from cucurbitadienol with the addition of 1-9 hydroxyl groups, 1-3 carbonyl groups, an acetyl group and/or the creation of a double bond (listed to the left). To the right in the table the masses (as *m/z*-values) of the potential intermediates is listed, with the loss of a hydrogen atom ($[M\text{-}H]^-$) and a water molecule ($[M\text{-}H2O\text{-}H]^-$) and with the addition of a formate adduct ($[M+HCOO]^-$). Masses used for calculation: C, 12; H, 1,007825; O, 15,99492; hydroxyl group, 15,99492; carbonyl group, 13,97927; double bond, -2,01565; acetyl group, 42,01057; H_2O, 18,01057; formate adduct, 45.

Hydroxyl	Carbonyl	Double bond	Acetyl	Formula		$[M\text{-}H]^-$	$[M\text{-}H2O\text{-}H]^-$	$[M+HCOO]^-$
0	0	0	0	C30H50O1		425,3784	407,3678	471,3862
1	0	0	0	C30H50O2		441,3733	423,3627	487,3811
2	0	0	0	C30H50O3		457,3682	439,3576	503,3760
3	0	0	0	C30H50O4		473,3631	455,3526	519,3710
4	0	0	0	C30H50O5		489,3581	471,3475	535,3659
5	0	0	0	C30H50O6		505,3530	487,3424	551,3608
6	0	0	0	C30H50O7		521,3479	503,3373	567,3557
7	0	0	0	C30H50O8		537,3428	519,3322	583,3506
8	0	0	0	C30H50O9		553,3377	535,3272	599,3456
9	0	0	0	C30H50O10		569,3327	551,3221	615,3405
0	1	0	0	C30H48O2		439,3576	421,3471	485,3655
1	1	0	0	C30H48O3		455,3526	437,3420	501,3604
2	1	0	0	C30H48O4		471,3475	453,3369	517,3553
3	1	0	0	C30H48O5		487,3424	469,3318	533,3502
4	1	0	0	C30H48O6		503,3373	485,3268	549,3452
5	1	0	0	C30H48O7		519,3322	501,3217	565,3401
6	1	0	0	C30H48O8		535,3272	517,3166	581,3350
7	1	0	0	C30H48O9		551,3221	533,3115	597,3299
8	1	0	0	C30H48O10		567,3170	549,3064	613,3248
0	2	0	0	C30H46O3		453,3369	435,3263	499,3447
1	2	0	0	C30H46O4		469,3318	451,3213	515,3397
2	2	0	0	C30H46O5		485,3268	467,3162	531,3346
3	2	0	0	C30H46O6		501,3217	483,3111	547,3295
4	2	0	0	C30H46O7		517,3166	499,3060	563,3244
5	2	0	0	C30H46O8		533,3115	515,3009	579,3193
6	2	0	0	C30H46O9		549,3064	531,2959	595,3143
7	2	0	0	C30H46O10		565,3014	547,2908	611,3092
0	3	0	0	C30H44O4		467,3162	449,3056	513,3240
1	3	0	0	C30H44O5		483,3111	465,3005	529,3189
2	3	0	0	C30H44O6		499,3060	481,2955	545,3139
3	3	0	0	C30H44O7		515,3009	497,2904	561,3088
4	3	0	0	C30H44O8		531,2959	513,2853	577,3037
5	3	0	0	C30H44O9		547,2908	529,2802	593,2986
6	3	0	0	C30H44O10		563,2857	545,2751	609,2935
0	0	1	0	C30H48O1		423,3627	405,3522	469,3706
1	0	1	0	C30H48O2		439,3576	421,3471	485,3655
2	0	1	0	C30H48O3		455,3526	437,3420	501,3604
3	0	1	0	C30H48O4		471,3475	453,3369	517,3553
4	0	1	0	C30H48O5		487,3424	469,3318	533,3502
5	0	1	0	C30H48O6		503,3373	485,3268	549,3452
6	0	1	0	C30H48O7		519,3322	501,3217	565,3401
7	0	1	0	C30H48O8		535,3272	517,3166	581,3350
8	0	1	0	C30H48O9		551,3221	533,3115	597,3299
9	0	1	0	C30H48O10		567,3170	549,3064	613,3248
0	1	1	0	C30H46O2		437,3420	419,3314	483,3498
1	1	1	0	C30H46O3		453,3369	435,3263	499,3447
2	1	1	0	C30H46O4		469,3318	451,3213	515,3397
3	1	1	0	C30H46O5		485,3268	467,3162	531,3346

4	1	1	0	C30H46O6		501,3217	483,3111	547,3295
5	1	1	0	C30H46O7		517,3166	499,3060	563,3244
6	1	1	0	C30H46O8		533,3115	515,3009	579,3193
7	1	1	0	C30H46O9		549,3064	531,2959	595,3143
8	1	1	0	C30H46O10		565,3014	547,2908	611,3092
0	2	1	0	C30H44O3		451,3213	433,3107	497,3291
1	2	1	0	C30H44O4		467,3162	449,3056	513,3240
2	2	1	0	C30H44O5		483,3111	465,3005	529,3189
3	2	1	0	C30H44O6		499,3060	481,2955	545,3139
4	2	1	0	C30H44O7		515,3009	497,2904	561,3088
5	2	1	0	C30H44O8		531,2959	513,2853	577,3037
6	2	1	0	C30H44O9		547,2908	529,2802	593,2986
7	2	1	0	C30H44O10		563,2857	545,2751	609,2935
0	3	1	0	C30H42O4		465,3005	447,2900	511,3084
1	3	1	0	C30H42O5		481,2955	463,2849	527,3033
2	3	1	0	C30H42O6		497,2904	479,2798	543,2982
3	3	1	0	C30H42O7		513,2853	495,2747	559,2931
4	3	1	0	C30H42O8		529,2802	511,2696	575,2880
5	3	1	0	C30H42O9		545,2751	527,2646	591,2830
6	3	1	0	C30H42O10		561,2701	543,2595	607,2779
0	0	0	1	C32H52O2		467,3889	449,3784	513,3968
1	0	0	1	C32H52O3		483,3839	465,3733	529,3917
2	0	0	1	C32H52O4		499,3788	481,3682	545,3866
3	0	0	1	C32H52O5		515,3737	497,3631	561,3815
4	0	0	1	C32H52O6		531,3686	513,3581	577,3765
5	0	0	1	C32H52O7		547,3635	529,3530	593,3714
6	0	0	1	C32H52O8		563,3585	545,3479	609,3663
7	0	0	1	C32H52O9		579,3534	561,3428	625,3612
8	0	0	1	C32H52O10		595,3483	577,3377	641,3561
9	0	0	1	C32H52O11		611,3432	593,3327	657,3511
0	1	0	1	C32H50O3		481,3682	463,3576	527,3760
1	1	0	1	C32H50O4		497,3631	479,3526	543,3710
2	1	0	1	C32H50O5		513,3581	495,3475	559,3659
3	1	0	1	C32H50O6		529,3530	511,3424	575,3608
4	1	0	1	C32H50O7		545,3479	527,3373	591,3557
5	1	0	1	C32H50O8		561,3428	543,3322	607,3506
6	1	0	1	C32H50O9		577,3377	559,3272	623,3456
7	1	0	1	C32H50O10		593,3327	575,3221	639,3405
8	1	0	1	C32H50O11		609,3276	591,3170	655,3354
0	2	0	1	C32H48O4		495,3475	477,3369	541,3553
1	2	0	1	C32H48O5		511,3424	493,3318	557,3502
2	2	0	1	C32H48O6		527,3373	509,3268	573,3452
3	2	0	1	C32H48O7		543,3322	525,3217	589,3401
4	2	0	1	C32H48O8		559,3272	541,3166	605,3350
5	2	0	1	C32H48O9		575,3221	557,3115	621,3299
6	2	0	1	C32H48O10		591,3170	573,3064	637,3248
7	2	0	1	C32H48O11		607,3119	589,3014	653,3198
0	3	0	1	C32H46O5		509,3268	491,3162	555,3346
1	3	0	1	C32H46O6		525,3217	507,3111	571,3295
2	3	0	1	C32H46O7		541,3166	523,3060	587,3244
3	3	0	1	C32H46O8		557,3115	539,3009	603,3193
4	3	0	1	C32H46O9		573,3064	555,2959	619,3143
5	3	0	1	C32H46O10		589,3014	571,2908	635,3092
6	3	0	1	C32H46O11		605,2963	587,2857	651,3041
0	0	1	1	C32H50O2		465,3733	447,3627	511,3811
1	0	1	1	C32H50O3		481,3682	463,3576	527,3760
2	0	1	1	C32H50O4		497,3631	479,3526	543,3710
3	0	1	1	C32H50O5		513,3581	495,3475	559,3659
4	0	1	1	C32H50O6		529,3530	511,3424	575,3608

5	0	1	1	C32H50O7		545,3479	527,3373	591,3557
6	0	1	1	C32H50O8		561,3428	543,3322	607,3506
7	0	1	1	C32H50O9		577,3377	559,3272	623,3456
8	0	1	1	C32H50O10		593,3327	575,3221	639,3405
9	0	1	1	C32H50O11		609,3276	591,3170	655,3354
0	1	1	1	C32H48O3		479,3526	461,3420	525,3604
1	1	1	1	C32H48O4		495,3475	477,3369	541,3553
2	1	1	1	C32H48O5		511,3424	493,3318	557,3502
3	1	1	1	C32H48O6		527,3373	509,3268	573,3452
4	1	1	1	C32H48O7		543,3322	525,3217	589,3401
5	1	1	1	C32H48O8		559,3272	541,3166	605,3350
6	1	1	1	C32H48O9		575,3221	557,3115	621,3299
7	1	1	1	C32H48O10		591,3170	573,3064	637,3248
8	1	1	1	C32H48O11		607,3119	589,3014	653,3198
0	2	1	1	C32H46O4		493,3318	475,3213	539,3397
1	2	1	1	C32H46O5		509,3268	491,3162	555,3346
2	2	1	1	C32H46O6		525,3217	507,3111	571,3295
3	2	1	1	C32H46O7		541,3166	523,3060	587,3244
4	2	1	1	C32H46O8		557,3115	539,3009	603,3193
5	2	1	1	C32H46O9		573,3064	555,2959	619,3143
6	2	1	1	C32H46O10		589,3014	571,2908	635,3092
7	2	1	1	C32H46O11		605,2963	587,2857	651,3041
0	3	1	1	C32H44O5		507,3111	489,3005	553,3189
1	3	1	1	C32H44O6		523,3060	505,2955	569,3139
2	3	1	1	C32H44O7		539,3009	521,2904	585,3088
3	3	1	1	C32H44O8		555,2959	537,2853	601,3037
4	3	1	1	C32H44O9		571,2908	553,2802	617,2986
5	3	1	1	C32H44O10		587,2857	569,2751	633,2935
6	3	1	1	C32H44O11		603,2806	585,2701	649,2885
